# Identification of a Locus Controlling Compound Raceme Inflorescence in Mungbean [*Vigna radiata* (L.) R. Wilczek]

**DOI:** 10.3389/fgene.2021.642518

**Published:** 2021-03-08

**Authors:** Eunsoo Lee, Xuefei Yang, Jungmin Ha, Moon Young Kim, Keum Yong Park, Suk-Ha Lee

**Affiliations:** ^1^Department of Agriculture, Forestry and Bioresources and Research Institute of Agriculture and Life Sciences, Seoul National University, Seoul, South Korea; ^2^Plant Genomics and Breeding Institute, Seoul National University, Seoul, South Korea; ^3^Department of Plant Science, Gangneung-Wonju National University, Gangneung, South Korea

**Keywords:** B3 transcription factor, compound raceme, inflorescence, legume, mungbean, synchronous maturity

## Abstract

Mungbean [*Vigna radiata* (L.) R. Wilczek] produces a compound raceme inflorescence that branches into secondary inflorescences, which produce flowers. This architecture results in the less-domesticated traits of asynchronous pod maturity and multiple harvest times. This study identified the genetic factors responsible for the compound raceme of mungbean, providing a unique biological opportunity to improve simultaneous flowering. Using a recombinant inbred line (RIL) population derived from VC1973A, an elite cultivar with a compound raceme type, and IT208075, a natural mutant with a simple raceme type, a single locus that determined the inflorescence type was identified based on 1:1 segregation ratio in the F_8_ generation, and designated *Comraceme*. Linkage map analysis showed *Comraceme* was located on chromosome 4 within a marker interval spanning 520 kb and containing 64 genes. RILs carrying heterozygous fragments around *Comraceme* produced compound racemes, indicating this form was dominant to the simple raceme type. Quantitative trait loci related to plant architecture and inflorescence have been identified in genomic regions of soybean syntenic to *Comraceme*. In IT208075, 15 genes were present as distinct variants not observed in other landrace varieties or wild mungbean. These genes included *Vradi04g00002481*, a development-related gene encoding a B3 transcriptional factor. The upstream region of *Vradi04g00002481* differed between lines producing the simple and compound types of raceme. Expression of *Vradi04g00002481* was significantly lower at the early vegetative stage and higher at the early reproductive stage, in IT208075 than in VC1973A. *Vradi04g00002481* was therefore likely to determine inflorescence type in mungbean. Although further study is required to determine the functional mechanism, this finding provides valuable genetic information for understanding the architecture of the compound raceme in mungbean.

## Introduction

Mungbean [*Vigna radiata* (L.) R. Wilczek] is a diploid (2n = 2x = 22) warm-season legume that belongs to the tribe Phaseoleae within the subfamily of Papilionoideae and is cultivated widely across Asia (Shanmugasundaram et al., 2009). Mungbean is not only a highly nutritious crop, due to its high concentration of folate and iron, but also highly efficient during cultivation, when factors such as self-pollination, a short life-cycle, and nitrogen fixation are considered (Kim et al., 2015). Despite the economic importance of mungbean, several agronomical traits, including early flowering, synchronous pod maturity, yield, and suitability of plant type for mechanized harvest, remain to be improved. All these traits are regarded as part of a domestication syndrome. Linkage mapping of domestication-related traits of mungbean has been less intensive than in other leguminous crops including soybean (*Glycine max*) and chickpea (*Cicer arietinum*). Indeed, most mapping results reported in mungbean involve resistance to insects, for example, to bruchid beetles (Chen et al., 2013; Chotechung et al., 2016; Schafleitner et al., 2016; Kaewwongwal et al., 2017, 2020) or to diseases, including yellow mosaic virus (Chen et al., 2013; Kitsanachandee et al., 2013; Alam et al., 2014; Mathivathana et al., 2019), powdery mildew (Chankaew et al., 2013; Yundaeng et al., 2020) and Cercospora leaf spot (Chankaew et al., 2011; Yundaeng et al., 2021). Although quantitative trait loci (QTLs) associated with flowering and traits related to domestication have been identified, including 34 quantitative traits and 4 qualitative traits (Isemura et al., 2012; Hwang et al., 2017), studies of yield-related traits are still at an early stage as there is a lack of the background information required to develop varieties suited to modern cultivation.

**Figure 1:**
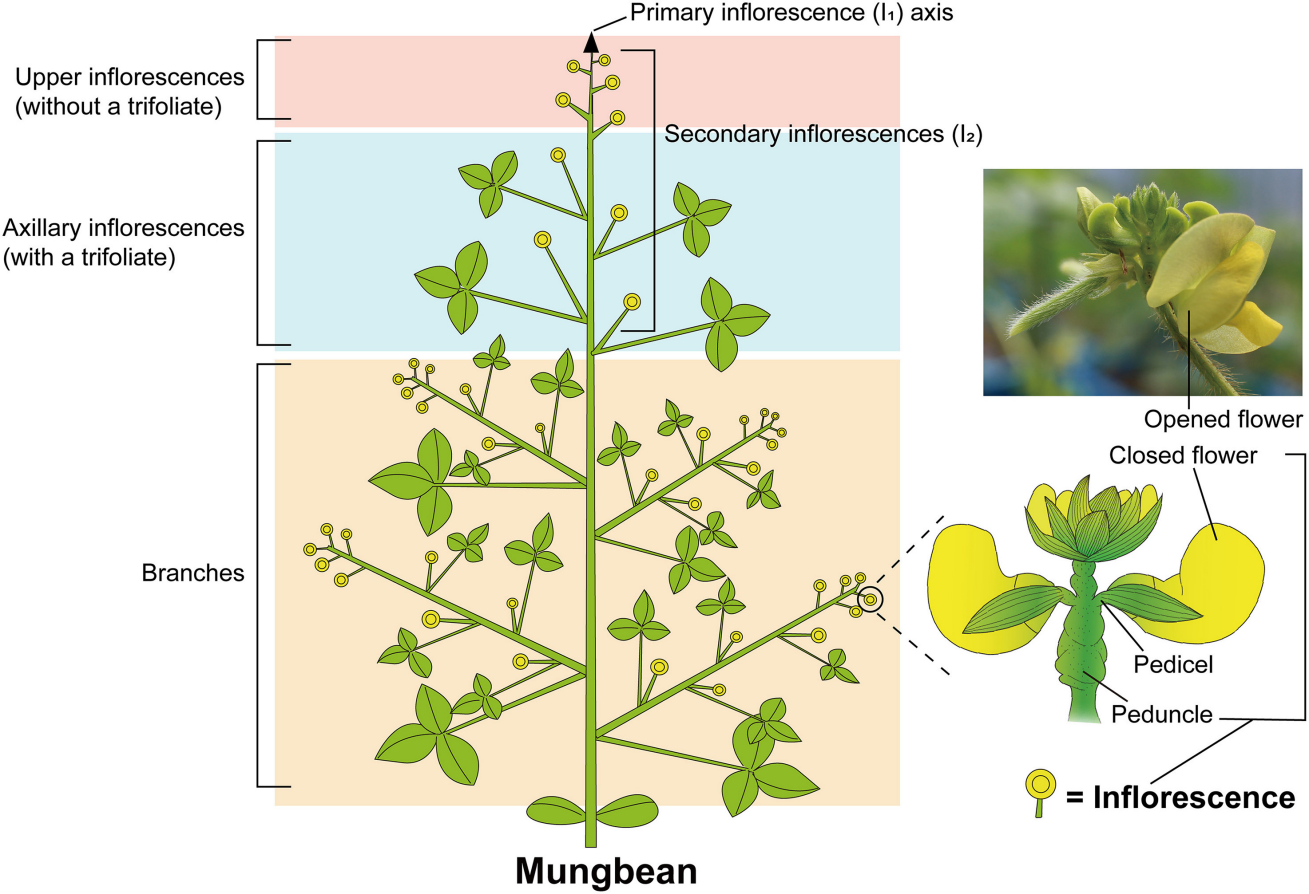
Schematic diagram of mungbean plant architecture. Mungbean shows a typical compound raceme that bears flowers not on the primary inflorescence (I_1_) but on secondary inflorescences (I_2_). Each branch in the lower part repeats the combination of axillary and upper inflorescences of the main stem. Double circles represent inflorescences; arrow indicates the primary indeterminate inflorescence axis.

Inflorescence architecture, which determines where the flowers form, determines the reproductive success and survival of plants, and is a result of evolution by natural selection (Prusinkiewicz et al., 2007); during domestication, inflorescence architecture is a key factor affecting crop yield and harvesting method. Inflorescence architecture is classified according to the types of branches produced (for e.g., raceme, panicle, and cyme) and the patterns of flowering (indeterminate and determinate inflorescence) (Benlloch et al., 2007). The raceme is an archetypal example of indeterminate inflorescence in which the axis elongates continuously through indefinite growth of the apical meristem and flowers are laterally generated along the axis. The raceme may be divided into simple and compound forms depending on whether or not the primary inflorescence meristem directly produces the flowers (Han et al., 2014). The *Arabidopsis thaliana* inflorescence is a simple raceme, as the flowers are borne on the main stem of the primary inflorescence (Ungerer et al., 2002; Han et al., 2014). Leguminous plants typically produce compound racemes on which secondary (I_2_) or higher order inflorescences develop from the primary (I_1_) inflorescence and bear flowers (Figure 1). As the axis of primary inflorescence is elongated, secondary inflorescences are continuously generated laterally and bear flowers in succession (Figure 1). In contrast to the simple raceme, individual inflorescences of the mungbean compound raceme contain flowers of many different ages, with the oldest at the bottom and younger ones above (Benlloch et al., 2015). This compound raceme inflorescence architecture is shared by most cultivated mungbean varieties and their wild relative, *Vigna radiata* var. *sublobata* (IBPGR, 1985; Bernardo et al., 2018). The cultivated mungbean therefore retains more less-domesticated and agronomically undesirable traits associated with inflorescence development, such as asynchronous pod maturity and multiple harvesting times, than does the cultivated soybean. Unraveling the genetic factors that control raceme inflorescence development is therefore important, both for understanding the evolution of plant form (Benlloch et al., 2015) and improving key architectural traits in mungbean.

We report here a natural mungbean mutant that showed a simple raceme inflorescence (Korean gene bank accession no. IT208075). This accession was used as a parental line to develop a recombinant inbred line (RIL) population of VC1973A (compound raceme) × IT208075 (simple raceme). We investigated the inheritance pattern of the compound raceme inflorescence and used a single nucleotide polymorphism (SNP)-based genetic linkage map to identify a locus controlling raceme inflorescence type. In addition, we identified a candidate gene located in a locus associated with the compound raceme inflorescence.

## Materials and Methods

### Plant Materials and Trait Phenotyping

A RIL population consisting of 235 F_7_ lines was developed from a cross between VC1973A (compound raceme) and IT208075 (simple raceme) by advancing the F_2_ to the F_6_ generation using the single seed descent method. The maternal line, VC1973A, was an elite cultivar developed at the World Vegetable Center (previously known as the Asian Vegetable Research and Development Center, AVRDC) in 1982, which was used to build the mungbean reference genome (Kang et al., 2014). The paternal line, IT208075, was a local variety from Vietnam named as Binh khe D.X. and was obtained from the National Agrobiodiversity Center, Republic of Korea (http://genebank.rda.go.kr/).

The 235 RILs and the mapping parents, VC1973A and IT208075, were planted in field trials over 2 growing seasons (planting dates: June 25, 2018 and June 20, 2019) at Seoul National University Experiment Farm (37°16′ 14.6″ N, 126°59′ 19.9″ E). Individual plants were set out in rows with 15 cm intervals between each plant, and the distance between rows was set at 70 cm. The plants were grown under natural photoperiods of 11.5 to 14.5 h per day. The inflorescence type of each RIL and parental line was scored as a compound or simple raceme depending on the presence or absence of a secondary inflorescence on the upper part of the main stem during the pod maturity period; 3 replicates were scored for each line. The number of secondary inflorescences on the upper part without a trifoliate was recorded at the time of harvest and used as a quantitative trait.

### DNA Extraction, Resequencing, and Genotyping-by-Sequencing

Genomic DNA was extracted from healthy young leaves of IT208075 and the 235 F_7:8_ RILs using the GenoAll^®^ Exgene^TM^ Plant SV kit (GeneAll Biotechnology, Seoul, Korea). DNA quality was assessed by the 260/280 nm ratio using a Nanodrop 3000 spectrometer (Thermo Scientific, Wilmington, DE, USA). DNA was quantified using the Invitrogen Quant-iT PicoGreen^®^ dsDNA Assay kit (Life Technologies, Burlington, ON, Canada), and its concentration was adjusted to 20 ng/μL. IT208075 was re-sequenced with 9× sequencing depth on an Illumina HiSeq2000 platform (Illumina Inc., San Diego, CA, USA). The Illumina reads were deposited in the database of the National Center for Biotechnology Information (NCBI) under BioProject accession number PRJNA698712. The genotyping-by-sequencing (GBS) method was used to genotype the RILs (Elshire et al., 2011). After digestion with the restriction enzyme *ApeKI*, a GBS library was constructed following the procedure described previously (Hwang et al., 2017; Yoon et al., 2019); the barcode adapters used in this study are listed in Supplementary Table 1. Three separate libraries were constructed using pooling amplified DNA samples from 84 or 85 RILs for each library. Single-end sequencing of the GBS libraries was performed on 3 lanes of an Illumina HiSeq2000 instrument (Illumina Inc.).

### Sequence Analysis, Genetic Map Construction, and QTL Analysis

Paired-end reads of IT208075 and GBS reads were mapped onto the mungbean reference genome (cv. VC1973A, PRJNA560716) using BWA v0.7.15 (Li, 2013) after trimming low quality sequence, barcode adapter, and *ApeKI* overhang sequences from raw reads. SNPs were called by SAMtools v1.3 (Li et al., 2009) and VCFtools (Danecek et al., 2011) with the following filtering criteria: mapping quality ≥30, read depth ≥3, heterozygosity ≤10%, and missing genotypes ≤70%. We constructed two separate genetic linkage maps for qualitative/quantitative analysis using JoinMap v4.1 (Van Ooijen, 2006). To anchor the locus for the qualitative trait of inflorescence type to the genetic map, the phenotypic difference was used as a molecular marker for linkage grouping along with the identified SNPs, where the order of the markers was determined using a maximum likelihood (ML) method. To confirm the position of the marker associated with inflorescence type on the genetic map, an additional genetic map was constructed with only SNP markers using regression mapping methods for QTL analysis. In both linkage mappings, the Kosambi mapping function was employed to translate the recombination frequency into a map distance in centimorgans (cM). QTL analysis was performed using IciMapping v4.1 software (Meng et al., 2015) through the ICIM-ADD mapping method, and the LOD threshold was determined by 1,000 permutation tests at a significance level of 0.05. Allelic associations with phenotypic differences were explored using RILs mapped to the genomic region surrounding the locus associated with a compound raceme.

### Comparative Analysis of the Locus for Compound Raceme

Soybean synteny blocks were identified by searching the soybean reference genome (Wm82.a2.v1, https://phytozome.jgi.doe.gov/) through MCScanX using BLASTp with default parameters to provide a comparative analysis of the 3 Mb genomic region on chromosome 4 surrounding the locus associated with a compound raceme (Vr04:26,001,525 to Vr04:28,997,867 bp). QTLs, markers, and gene information for soybean were obtained from the SoyBase database (http://www.soybase.org). The plant transcription factor database PlantTFDB (http://planttfdb.gao-lab.org/) was used to obtain information about transcription factor genes (Jin et al., 2017). TAIR version 10 (http://www.arabidopsis.org) was searched using BLASTp to find orthologous genes of *A. thaliana*.

### Identification of Sequence Variation in Genes Within the Compound Raceme Locus

We investigated sequence variation between VC1973A and IT208075 in genes located in the locus associated with the compound raceme to identify likely candidate genes responsible for this phenotype. The variant sequences were compared with previously reported sequences of two other mungbean lines producing compound racemes, TC1966 (wild mungbean, *Vigna radiata var. sublobata*) and V2984 (a Korean landrace), to identify sequence variation specific to IT208075, which has a simple raceme (NCBI accession code JJMO00000000, Kang et al., 2014). We used Sanger sequencing to validate the sequence variation in two promising genes, *VrDet1* and *Vradi04g00002481*. PCR products, amplified with sequence-specific primers (Supplementary Table 2), were purified from 1% agarose gels using the AccuPrep^®^ Gel Purification Kit (Bioneer, Daejon, Korea), cloned into *Escherichia coli* using the pGEM-T Easy Vector (Promega, Madison, WI, USA), according to the manufacturer's instructions, and sequenced using an ABI 3730XL DNA analyzer (Applied Biosystems, Foster City, CA, USA).

### Motif Discovery and qRT-PCR Analysis of Selected Genes

To determine the domain conserved between mungbean and *A. thaliana* orthologs, sequences were aligned using the MEME suite motif analysis tool (Bailey et al., 2009). Differences in expression levels of selected genes between the mapping parents were compared using newly emerged shoot apical tissue (SAT) collected at each developmental stage. Unifoliate opening marked the first developmental stage. The SAT of each subsequent developmental stage was sampled when the second uppermost trifoliate leaflet of the node had fully opened; as VC1973A had 8 nodes and IT208075 had 4 nodes, they consisted of 9 and 5 developmental stages, respectively. Three biological samples were analyzed at each developmental stage, and one biological sample consisted of SAT from 5 individual plants exhibiting the same developmental stage. Total RNA was extracted from each sample using a Ribospin^TM^ Plant kit (GeneAll Biotechnology, Seoul, Korea). cDNA was synthesized using a Bio-Rad iScript^TM^ cDNA Synthesis Kit (Bio-Rad, Hercules, CA, USA). Primer sequences for qRT-PCR were designed using Primer3 (http://bioinfo.ut.ee/primer3-0.4.0/) and are listed in Supplementary Table 2. qRT-PCR was performed with 2 technical replicates using the Bio-Rad iQ^TM^ SYBR Green Supermix Kit in a LightCycler^®^ 480 (Roche Diagnostics, Laval, QC, Canada). The amplification conditions were 95°C for 5 min, followed by 40 cycles of 95°C for 10 s, 60°C for 15 s, and 72°C for 15 s. *eIF5A* (*Vradi05g00001056*), which encodes eukaryotic initiation factor 5A, was used as a reference gene for normalizing expression levels of target genes, and relative expression of each gene was calculated according to the 2^−ΔΔ^CT method (Livak and Schmittgen, 2001). Statistical significance was determined using the Student's *t*-test.

## Results

### A Simple Raceme Inflorescence Was Observed in IT208075

**Figure 2:**
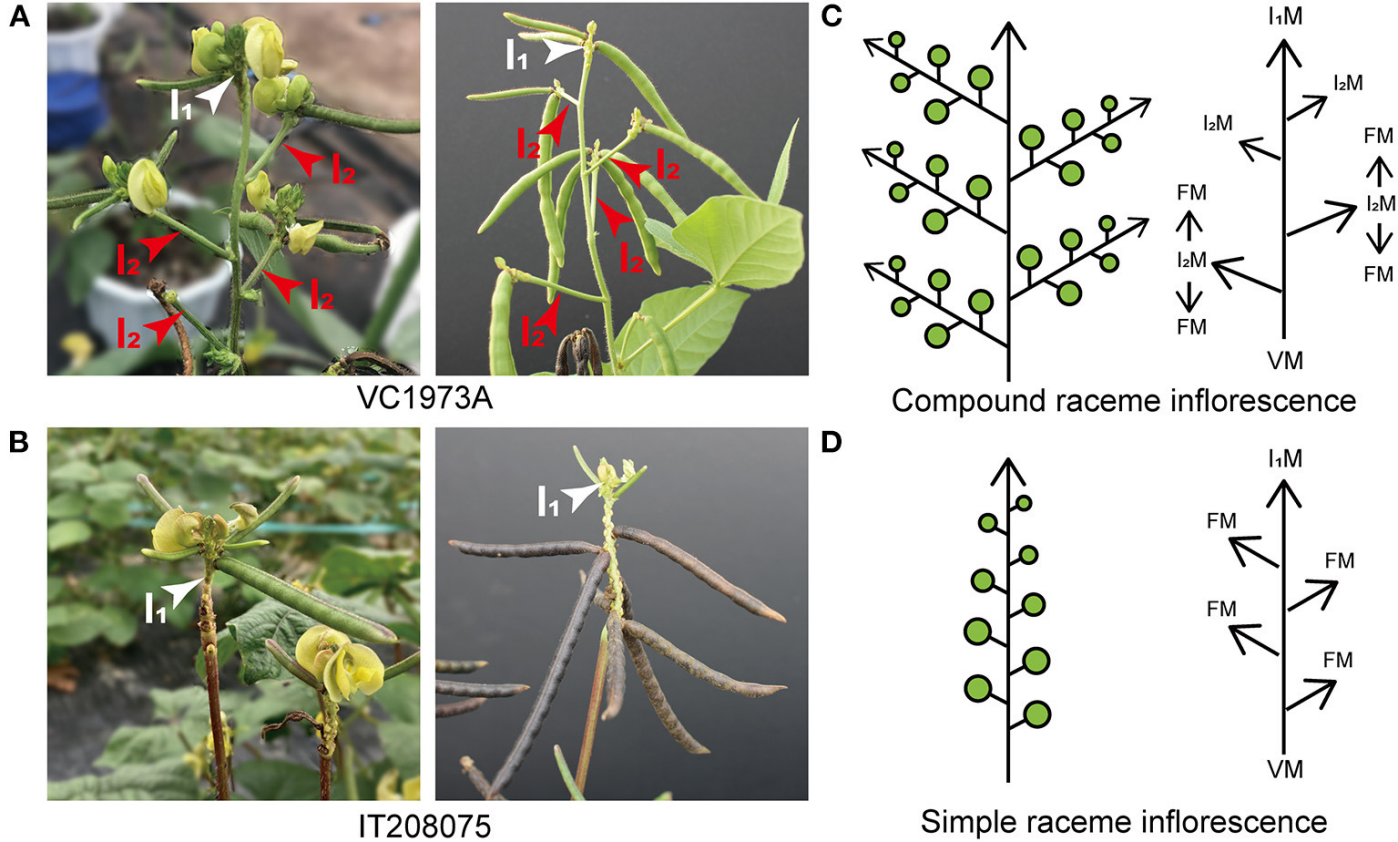
Morphological differences in upper inflorescences between the parental lines VC1973A and IT208075. **(A)** VC1973A compound raceme (left photograph) and podding (right photograph). **(B)** IT208075 simple raceme (left photograph) and podding (right photograph). **(C)** Diagram showing compound raceme and meristem development. In the shoot apical meristem, the vegetative meristem (VM) is transferred to the primary inflorescence meristem (I_1_M), which then generates the secondary inflorescence meristem (I_2_M) to produce the floral meristem (FM). Flowers are borne along the secondary inflorescence (I_2_) axes. **(D)** Diagram showing simple raceme and meristem development. Flowers are borne along the primary inflorescence (I_1_) without generating secondary inflorescences. **(A,B)** White arrows indicate primary inflorescences; red arrows indicate secondary inflorescences. **(C,D)** Green circles represent flowers and pods.

The maternal line, VC1973A, had a typical compound raceme. It showed indeterminate flowering, and thus an individual inflorescence produced different cohorts of flowers, with the oldest ones located at the bottom and younger ones developing consecutively toward the top (Figure 2A). Consequently, the pods matured at different times, and the same inflorescence simultaneously carried mature pods and new flowers. We identified a landrace variety, IT208075, which did not produce secondary inflorescences on the upper part of its main stem. Instead, a single primary inflorescence was transferred from the axis of the main stem and bore flowers in an acropetal succession (Figure 2B). This architecture is defined as a simple raceme inflorescence, as typified by the model plant *A. thaliana*.

### Inheritance of the Compound Raceme Inflorescence

A phenotypic investigation was conducted in a RIL population derived from a cross between VC1973A and IT208075 to identify the pattern of genetic inheritance underlying the inflorescence architecture of mungbean. The mean numbers of upper inflorescences produced by VC1973A were 7.0 and 4.7 in 2018 and 2019, respectively, whereas IT208075 produced a single primary inflorescence (Table 1). Within the population of 235 F_8:9_ RILs, 122 produced a compound raceme, 99 produced a simple raceme, and 14 lines showed a segregating phenotype (Supplementary Table 3). A Chi-square test indicated that these observations were consistent with segregation according to a 1:1 Mendelian ratio (*X*^2^ = 2.4, *P* = 0.12), suggesting that a single gene, designated *Comraceme*, controlled the compound raceme phenotype in VC1973A.

**Table 1:** Phenotypic variation and heritability (*H*^2^) of the number of upper inflorescences in VC1973A and IT208075 and their derived RIL population.

**Year**	**Parental lines ± SD**			**RIL population**				
	**VC1973A**	**IT208075**	***P-value***	**Average**	**SD**	**Min**	**Max**	***H*^2^^a^**
2018	7.0 ± 1.0	1.0	0.0091^**^	3.6	2.8	1.0	8.3	0.94
2019	4.7 ± 0.3	1.0	0.0081^**^	2.5	1.5	1.0	6.0	0.98

a *Broad-sense heritability*.

** *Significance at the 0.01 probability level; SD, standard deviation*.

### Resequencing and GBS Analysis

In total, 4.3 Gb raw data from 31 million reads were generated from IT208075 (Supplementary Table 4). After quality trimming, 23 million high quality reads were mapped against the mungbean reference genome, which resulted in 91.2% genome coverage and 7.7× mapping depth. To genotype the RIL population, a total of 672 million GBS reads of 235 RILs were mapped to the reference genome. The number of GBS reads mapped to the reference genome ranged from 1,202,609 to 6,565,325 (mean: 2,680,760 reads). The percentage coverage of the genome ranged from 1.3 to 3.4% (mean: 2.2%). A total of 4,177 SNPs was used to construct genetic maps.

### Mapping of the *Comraceme* Locus

**Figure 3:**
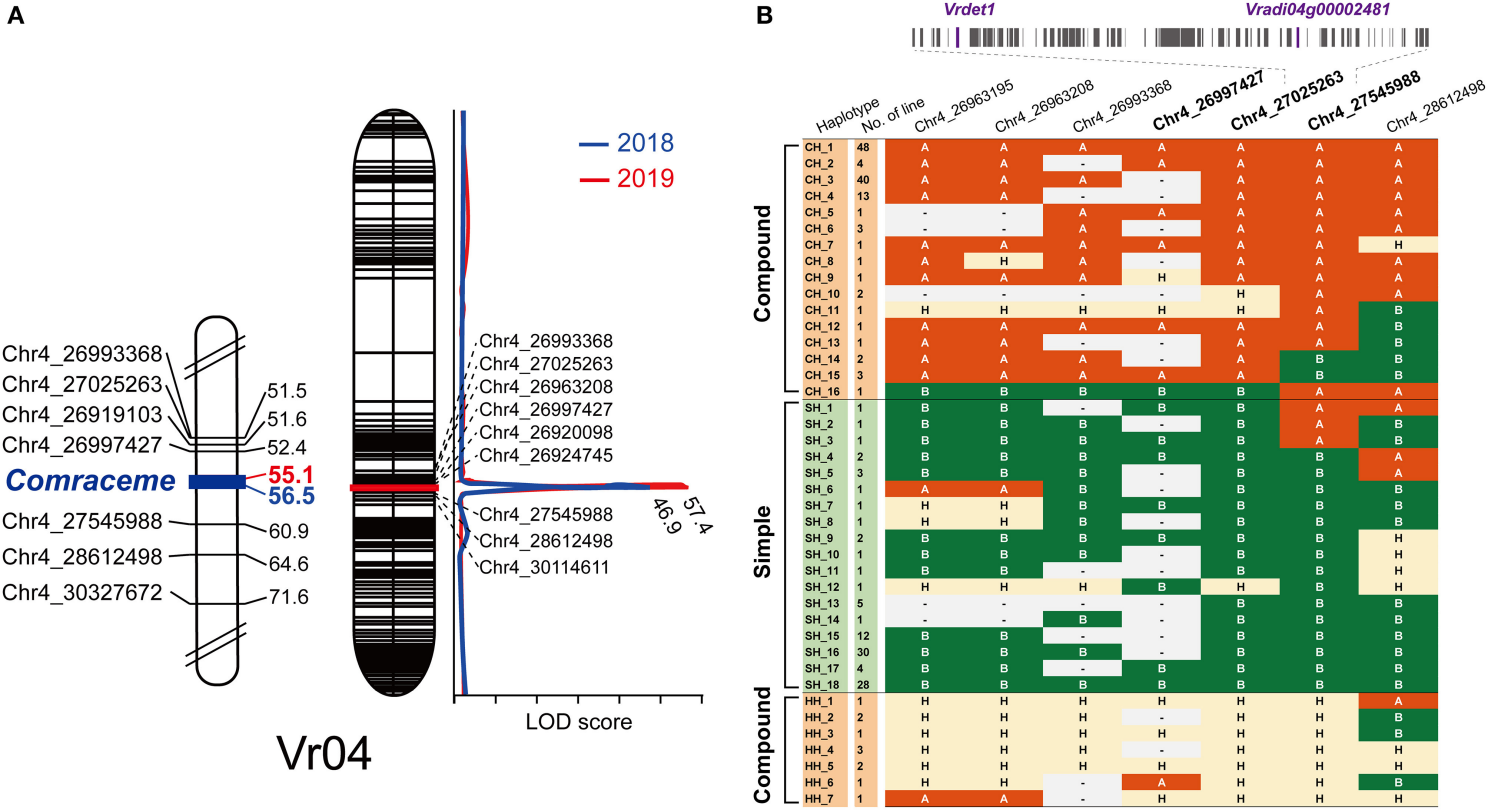
Genetic mapping of the compound raceme of mungbean. **(A)** The position of the *Comraceme* locus for compound raceme. *Comraceme* was identified separately as a qualitative (left) and quantitative (right) trait using data collected during 2018 and 2019. It was located between markers Chr4_26997427 and Chr4_27545988 (left) and Chr4_26924745 and Chr4_27545988 (right). **(B)** SNP haplotypes around the *Comraceme* locus, the number of RILs by haplotype, and their phenotypical associations. Based on the phenotypic association of 3 haplotypes, CH_15, CH_16, and SH_1, the recombination break point was inferred to occur between Chr4_27025263 and Chr4_27545988, which corresponded to a physical length of 520.1 Kb. RILs heterozygous for alleles around the *Comraceme* locus (haplotypes HH_1 to 7) showed the compound raceme type. The 64 protein-coding genes between the markers flanking the *Comraceme* locus are shown as small gray rectangles; *Vrdet1* (*Vradi04g00002442*) and *Vradi04g00002481* are shown in purple. A (orange background): VC1973A allele; B (green background): IT208075 allele; H (yellow background): heterozygous allele.

To locate *Comraceme*, the locus associated with the qualitative raceme trait, we constructed a genetic map containing phenotypic data of compound/single raceme type as a molecular marker among the RIL population. Of the 4,177 SNP markers available, the final genetic map contained 1,799 SNPs over 11 chromosomes (Vr01 to Vr11) and spanned 1,353.5 cM with a mean marker interval of 0.82 cM by the ML method (Supplementary Table 5). The number of SNPs mapped to each chromosome ranged between 54 (Vr07) and 357 (Vr02) with a mean of 164. *Comraceme* was located in the interval between markers Chr4_26997427 and Chr4_27545988, which spanned 545.6 Kb of Vr04 (Table 2, Figure 3A).

**Table 2:** Genetic mapping for the compound raceme inflorescence in the RIL population derived from VC1973A × IT208075.

**Phenotyping**	**Year**	**Chr**	**Pos^a^ (cM)**	**Marker interval**	**LOD^b^**	**PVE^c^ (%)**	**Add^d^**	**No. of genes^e^**
Qualitative	2018	4	55.1	chr4_26997427:chr4_27545988				67
	2019	4	56.5	chr4_26997427:chr4_27545988				67
Quantitative	2018	4	15.0	chr4_26924745:chr4_27545988	46.9	62.0	−2.1	73
	2019	4	15.0	chr4_26924745:chr4_27545988	57.4	67.6	−1.2	73

a *Mapped position of a qualitative trait as a marker in the genetic linkage map following ML mapping method, and genetic position of a QTL peak in the genetic linkage map constructed following regression mapping method*.

b *Maximum-likelihood logarithm of odds (LOD) score for the individual QTL*.

c *Percent of phenotypic variance explained by the QTL*.

d *Allelic additive effect*.

e *Number of protein-coding genes within marker intervals*.

To validate *Comraceme*, composite interval mapping was conducted using the number of the upper inflorescences as the quantitative trait. RILs producing a simple raceme were recorded as having one inflorescence. The additional genetic map spanning 450.1 cM was constructed using the regression mapping method (Supplementary Figure 1, Supplementary Table 6). The mean interval between markers was 0.33 cM, although it ranged from 0.06 cM on Vr10 to 0.77 cM on Vr11 (Supplementary Table 5). A major QTL was detected between Chr4_26924745 and Chr4_27545988 that exceeded the significance level on Vr04 for the number of upper inflorescences in both the 2018 and 2019 datasets. This QTL had LOD scores of 46.9 (2018 data) and 57.4 (2019 data), which explained 62.0 and 67.6% of the phenotypic variation, respectively (Table 2, Figure 3A). Both qualitative and quantitative analyses of the compound raceme inflorescence trait identified the marker Chr4_27545988 as a boundary of the interval containing the *Comraceme* locus (Table 2).

We compared the phenotypes of RILs with different haplotypes around the *Comraceme* locus to delimit the position of *Comraceme*. The order of the marker Chr4_27025263 in the genetic maps differed from its physical position in the reference genome between Chr4_26997427 and Chr4_27545988, probably because of insufficient genotype data (Figures 3A,B). Although haplotypes CH_15 and CH_16 had different alleles at Chr4_27025263 and Chr4_27545988, both produced the compound raceme inflorescence phenotype. By contrast, haplotypes CH_16 and SH_1 had the same alleles at both marker positions but produced compound and simple raceme inflorescences, respectively. This suggested the recombination break occurred between Chr4_27025263 and Chr4_27545988, corresponding to a physical distance of 520.1 Kb. In addition, 11 RILs carrying a heterozygous fragment around the *Comraceme* locus produced a compound raceme, indicating that a single, dominant gene controlled *Comraceme* and was responsible for the compound raceme phenotype.

### Syntenic Analysis of the *Comraceme* Locus and the Soybean Genome

**Figure 4:**
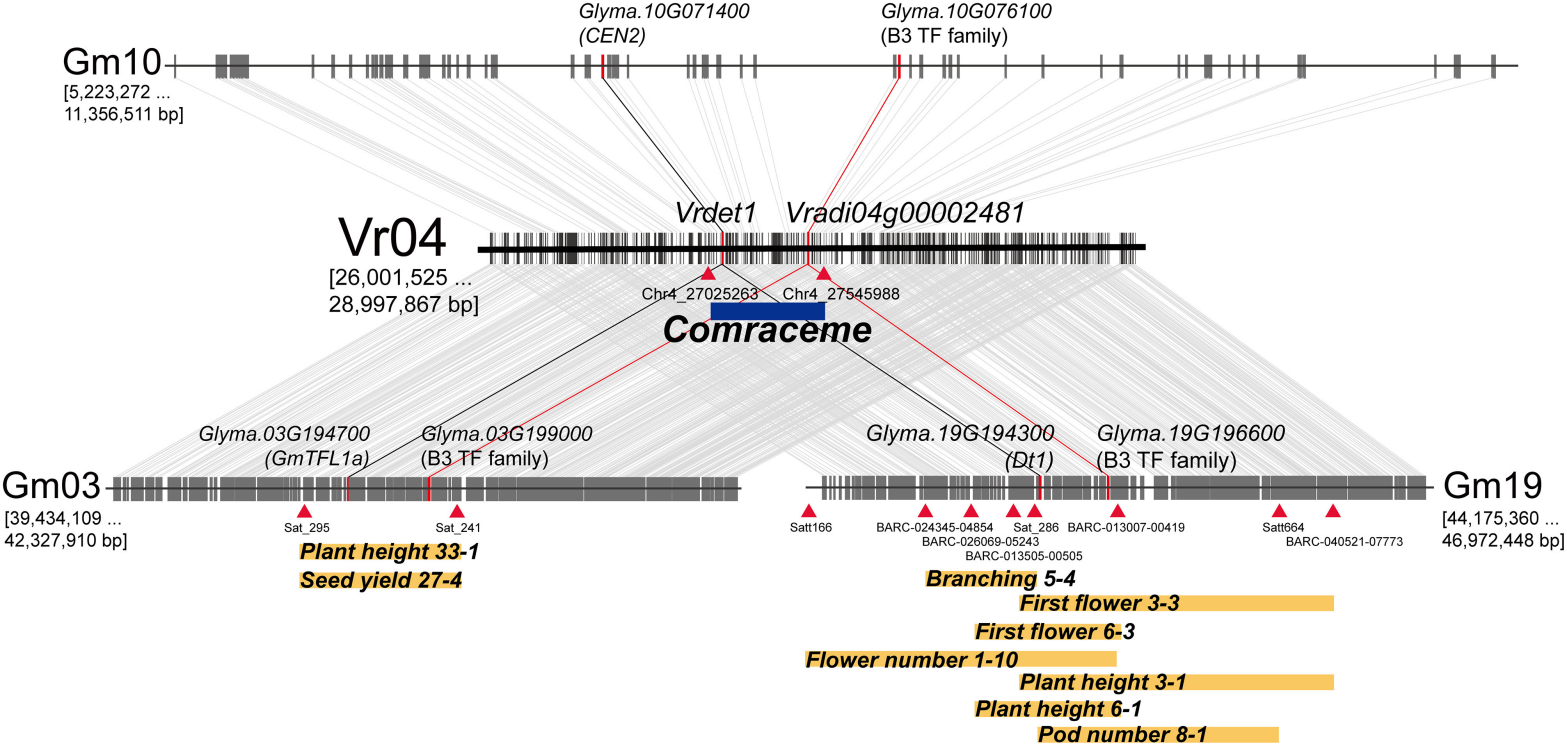
Soybean genomic blocks syntenic with the 3 Mb genomic region of mungbean harboring the *Comraceme* locus (Vr04: 26,001,525 to 28,997,867 bp). The soybean syntenic blocks were physically located on 3 chromosomes, Gm03 (39,434,109 to 42,327,910 bp); Gm10 (5,223,272 to 11,356,511 bp); and Gm19 (44,175,360 to 46,972,448 bp). The syntenic blocks on Gm03 and Gm19 contain previously known QTLs associated with inflorescence-related agronomic traits. *Dt1* (*Glyma.19G194300*) and *VrDet1*, which are responsible for determinate vegetative growth in soybean and mungbean, respectively, also show conserved colinearity. Other homologous genes of soybean, *GmTFL1a* (*Glyma.03G194700*) and *CEN2* (*Glyma.10G071400*), are also present on Gm03 and Gm10, respectively. The genes *Vradi04g00002481, Glyma.03G199000, Glyma.10G076100*, and *Glyma.19G196600*, which encode proteins of the B3 TF subfamily, are maintained in the genomic blocks syntenic between mungbean and soybean; changes to the transcriptional regulation of these genes cause pleiotropic growth defects in plant development. Blue and yellow rectangles represent QTL intervals; red triangles indicate markers flanking the QTLs.

As soybean is the closest model legume species to mungbean, we analyzed the syntenic relationship between the genomic region containing *Comraceme* and the soybean genome. A comparison between the 3 Mb mungbean genomic region containing the 520 Kb segment between Chr4_27025263 and Chr4_27545988 and the soybean genome identified 3 colinear blocks, located on the soybean chromosomes Gm03, Gm10, and Gm 19. The syntenic regions located on Gm03 and Gm19 contained soybean QTLs associated with inflorescence-related agronomic traits, including plant height, branching, first flower, flower number, pod number, and seed yield (Lark et al., 1995; Mansur et al., 1996; Orf et al., 1999; Zhang et al., 2010; Kim et al., 2012; Kuroda et al., 2013; Shim et al., 2017). The soybean QTL *Plant height 33–1* and *Seed yield 27–4* were located on Gm03, whereas *Branching 5–4*, First *flower 3–3* and *6–3, Flower number 1–10, Plant height 3–1* and *6–1*, and *Pod number 8–1* were all located on Gm19 (Figure 4). In addition, the syntenic blocks on Gm03 and Gm19 contained *GmTFL1a* and *GmDt1* (*GmTFL1b*), respectively; both are orthologs of *Arabidopsis TERMINAL FLOWER 1* (*TFL1)*. *VrDet1*, the ortholog of *GmDt1*, was also located in the *Comraceme* locus. *GmDt1* and *VrDet1* modulate determinate stem growth habit in soybean and mungbean, respectively (Liu et al., 2010; Tian et al., 2010; Li et al., 2018). No QTL associated with inflorescence-related traits were located in the syntenic region of Gm10, although this region did contain *GmCEN2*, a homolog of *AtTFL1*.

### Identification of a Gene Candidate for *Comraceme*

**Figure 5:**
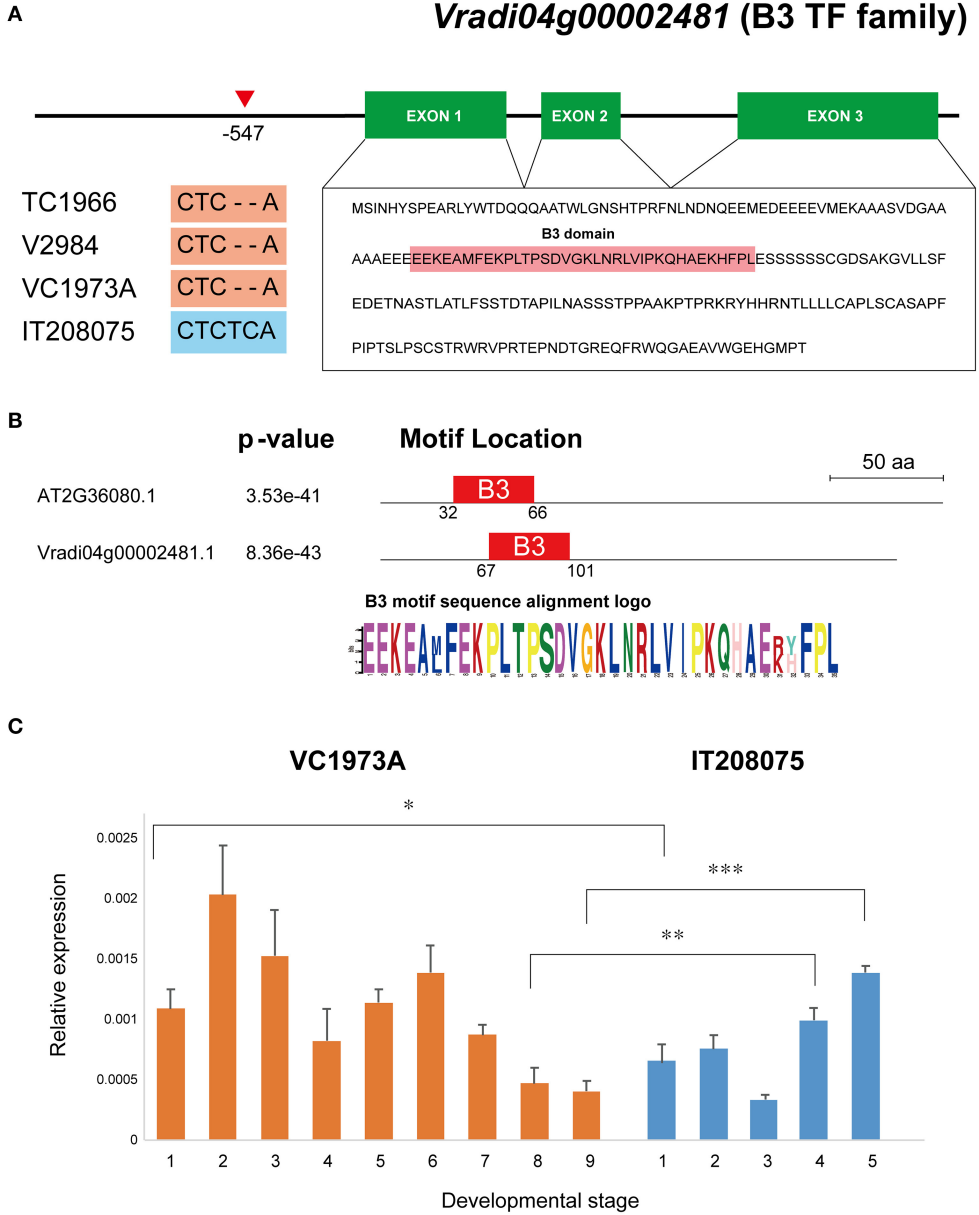
Gene structure and expression level of *Vradi04g00002481*, which encodes a B3 TF family protein. **(A)** Two-base insertion specific to IT208075 located −547 bp upstream of the coding region of the gene. Three mungbean lines producing compound racemes, VC1973A, V2984, and TC1966, showed a two-base deletion at this point, indicated by a red triangle. *Vradi04g00002481* consists of 3 exons encoding 224 amino acids and contains a B3 domain. **(B)** B3 motif sequence alignment between AT2G36080 and Vradi04g00002481. **(C)** Expression levels of *Vradi04g00002481* in shoot apical tissue from lines VC1973A and IT208075 at each developmental stage. *P < 0.05; **P < 0.01; ***P < 0.001.

The *Comraceme* locus between Chr4_27025263 and Chr4_27545988 contained 64 protein-coding genes, 55 of which have functionally annotated *A. thaliana* orthologs (Supplementary Table 7). After comparing all the variant sequences found in the *Comraceme* genomic region with those known from *Vigna* varieties with a compound raceme (the landrace V2984 and the wild mungbean TC1966), we identified 15 genes with variants present only in the IT208075 paternal line, which produced a simple raceme inflorescence. Among these genes, 2 genes, *VrDet1* and *Vradi04g00002481* were likely to affect inflorescence type based on previous studies demonstrating their roles in growth and development (Supplementary Table 8). *VrDet1* modulates the stem growth habit of mungbean (Li et al., 2018). The first intron region of *VrDet1* showed variation of simple repeat sequences (SSRs) [(AC)n(AT)n] between lines: TC1966 had a 4 bp insertion of SSRs (“AC” and “AT”), IT208075 had a 2 bp deletion (“AC”), whereas VC1973A and V2984 had the same sequence (Supplementary Table 8, Supplementary Figure 2A). The mapping parents also differed with respect to two insertion-deletion mutations (indels) in the upstream and second intron regions of *VrDet1* but these mutations were not detected in TC1966 and V2984 owing to unmapped gaps (Supplementary Figure 2A). *Vradi04g00002481* is a homolog of an *A. thaliana* gene, *ABNORMAL SHOOT 2* (*ABS2*, also named *NGATHA-Like 1*, AT2G36080), which causes pleiotropic growth defects (Shao et al., 2012). IT208075 showed specific sequence variation of two-base insertion (“TC”) in the upstream region of *Vradi04g00002481*, whereas TC1966, V2984, and VC1973A had the same sequence (Figure 5A). The predicted Vradi04g00002481 protein consisted of 224 residues and included a B3 DNA-binding domain that showed a high similarity with the motif of ABS2 (Figure 5B).

### Expression Analysis During Mungbean Development

To determine whether transcription levels differed in the selected genes *VrDet1* and *Vradi04g00002481*, we used qRT-PCR to examine their expression level in the SAT at each developmental stage. We used the differences in plant architecture between the parental lines VC1973A and IT208075 to select 3 developmental stages in which the same organs were generated: the beginning stage when the trifoliate emerged (stage 1 for both accessions) after the unifoliate was fully opened; the terminal stage of the vegetative state when the final trifoliate emerged on the main stem (stage 8 for VC1973A and stage 4 for IT208075); and the early reproductive stage when the apical shoot was transformed to generate the peduncle for inflorescence (stage 9 for VC1973A and stage 5 for IT208075).

There were no significant differences in expression levels of *VrDet1* between the parental lines at any of the 3 developmental stages (Supplementary Figure 2B). By contrast, significant differences in the levels of *Vradi04g00002481* expression were observed: *Vradi04g00002481* expression in VC1973A was significantly higher than in IT208075 at the beginning stage (stage 1), but expression in IT208075 was significantly higher than in VC1973A at the other 2 stages (Figure 5C). These results indicated that transcription of *Vradi04g00002481* was more likely to affect the inflorescence architecture of mungbean than *VrDet1*.

## Discussion

The complex processes controlling inflorescence architecture formation significantly affect plant reproduction. A compound raceme inflorescence is defined by the presence of secondary inflorescences to produce flowers and is often observed in legume species in the Papilionoideae subfamily (Berbel et al., 2012; Han et al., 2014; Benlloch et al., 2015). Plants with a compound raceme generate peduncles from the axis of the main stem toward the direction of elongation; the peduncles become stalks, each supporting a raceme inflorescence and thus enabling constant blooming. This architecture, depicted in a simplified form in Figure 2C, is observed in the maternal line, VC1973A. The number of flowers per plant depends on the duration of activation of the apical meristem in the primary inflorescence (I_1_M) as this controls the indeterminate initiation of the secondary inflorescence meristems (I_2_M), which produce the floral meristems that generate flowers (Tucker, 2003; Benlloch et al., 2007). Plants with a simple raceme type, such as *Arabidopsis*, do not generate peduncles and produce flowers only from the axis of the main stem as the apical meristem develops into the floral meristem (Shannon and Meeks-Wagner, 1993; Bradley et al., 1997; Ungerer et al., 2002). This architecture is observed in the paternal line, IT208075, and is depicted in Figure 2D.

Relatively few studies of inflorescence architecture have been reported from mungbean compared with other major crops. Although inheritance of the inflorescence type, the number of clusters per node, and sterility have been investigated (Sen and Ghosh, 1959; Singh and Singh, 1970; Selvi et al., 2003; Mehandi et al., 2019), the genetic basis underlying inflorescence formation remains unexplained. In the current study, the compound and simple inflorescence types appeared in a 1:1 ratio among the RIL population, as would be expected for a qualitative trait controlled by a single gene. To map the gene controlling the qualitative trait, we treated the phenotypic data of RILs as a molecular marker following the alleles of parental lines (Isemura et al., 2012; Mei et al., 2017; Pereira et al., 2018). This showed that the *Comraceme* locus was on Vr04 and determined the inflorescence type. The number of upper inflorescences varied among the subset of the RIL population, even though they showed a compound inflorescence type. Analysis of the number of upper inflorescences as a quantitative trait identified a major QTL on Vr04 that shared a marker (Chr4_27545988) with *Comraceme*. Furthermore, we observed that a few RILs with heterozygous haplotypes around the *Comraceme* locus produced a compound raceme, and concluded that the type of inflorescence was controlled by a single dominant allele.

*Comraceme* was located in a 520.1 kb genomic region that contained 64 protein-coding genes, 15 of which had conserved nucleotide variants in IT208075 that were not observed in *Vigna* varieties with a compound raceme. We selected two of these genes, *Vrdet1* and *Vradi04g00002481*, as potential candidates for *Comraceme* and analyzed their expression level in the SAT between the parental lines. *VrDet1* is an ortholog of the inflorescence meristem identity gene *TFL1*, which together with two other floral meristem identity genes, *LEAFY* (*LFY*) and *APETAL1* (*AP1*), participates in the transition between the vegetative and reproductive stages in *Arabidopsis* (Shannon and Meeks-Wagner, 1993; Liljegren et al., 1999). In mungbean, the determinate growth habit is determined by a mutation in the promoter region of *Vrdet1* that regulates gene expression during the vegetative stage (Li et al., 2018). Although we identified a variant in the upstream region of *VrDet1* (Supplementary Figure 2A), there were no significant differences between the parental lines in *VrDet1* expression levels in the SATs throughout the vegetative and reproductive developmental stages (Supplementary Figure 2B). *Vradi04g00002481* is a homolog of *Arabidopsis ABS2* (AT2G36080) and belongs to a B3-plant specific transcription factor regulating plant growth and development. In *Arabidopsis, ABS2* encodes a transcription factor in the RAV subfamily, and its over-expression produces abnormal shoot and floral petal development as a pleiotropic effect (Shao et al., 2012). Transgenic *Medicago truncatula* plants over-expressing *MtRAV* exhibit increased bolting and bifurcation of sub-branches up to tertiary level (Wang et al., 2020). The soybean *E1* gene is one of the major flowering genes and has a genetic region resembling the B3 domain (Xia et al., 2012; Benlloch et al., 2015). In this context, it is notable that VC1973A had many more nodes and branches, produced more inflorescences, and showed a later flowering time than IT208075. *Vradi04g00002481* had one variant in the promoter region only from IT208075 (Figure 5A) and the significant differences in levels of *Vradi04g00002481* expression were observed between VC1973A and IT208075 (Figure 5C). These results suggested that Vradi04g00002481 functioned as a modulator that determined inflorescence architecture and was a key factor affecting flowering.

Many agronomically important traits, such as seed dormancy, 100-seed weight, plant type, and phenology-related traits, have been improved for the benefit of human beings during mungbean domestication (Isemura et al., 2012). In general, traits related to high yield and mechanical cultivation have been the main target of crop breeding programs. Several traits have been selected from wild and exotic species to produce modern cultivated varieties with a suitable plant architecture and high crop productivity. Such traits include vine to erect growth habit (Wang et al., 2019), stem termination (Tian et al., 2010), and pod shattering (Dong et al., 2014). In this study, we identified IT208075, a simple raceme genotype having a single primary inflorescence. This genotype is expected to show shorter flowering duration than VC1973A of a compound raceme, probably leading to higher synchronicity of pod maturity. IT208075 will be a useful breeding material for mungbean improvement, even though the correlation between the simple raceme inflorescence and other agricultural traits such as flowering duration, synchronous pod maturity and seed yield is yet to be elucidated. Furthermore, the linkage mapping and QTL analysis of a RIL population derived from a cross of VC1973A and IT208075 suggested that *Vradi04g00002481* was the candidate gene responsible for the formation of compound raceme inflorescence architecture. Although further study is required to determine the functional role played by this gene in modulating inflorescence type, these results broaden our understanding of the genetic background of inflorescence architecture in mungbean.

## Data Availability Statement

The datasets presented in this study can be found in online repositories. The name of the repository and accession number can be found at: National Center for Biotechnology Information (NCBI) BioProject, https://www.ncbi.nlm.nih.gov/bioproject/, PRJNA698712.

## Author Contributions

EL and S-HL conceived and designed the research. EL and KYP developed the RIL population. EL and XY conducted the experiments. EL analyzed the data. EL and MYK wrote the manuscript. MYK, JH, and S-HL revised the manuscript. S-HL supervised the project. All authors read and approved the manuscript.

## Conflict of Interest

The authors declare that the research was conducted in the absence of any commercial or financial relationships that could be construed as a potential conflict of interest.

## References

[B1] Alam A. K. M. M. Somta P. Srinives P. (2014). Identification and confirmation of quantitative trait loci controlling resistance to mungbean yellow mosaic disease in mungbean [*Vigna radiata* (L.) wilczek]. Mol. Breed. 34, 1497–1506. 10.1007/s11032-014-0133-0

[B2] Bailey T. L. Boden M. Buske F. A. Frith M. Grant C. E. Clementi L.. (2009). MEME suite: tools for motif discovery and searching. Nucleic Acids Res. 37, W202–W208. 10.1093/nar/gkp335 19458158 PMC2703892

[B3] Benlloch R. Berbel A. Ali L. Gohari G. Millán T. Madueño F. (2015). Genetic control of inflorescence architecture in legumes. Front. Plant Sci. 6:543. 10.3389/fpls.2015.00543 26257753 PMC4508509

[B4] Benlloch R. Berbel A. Serrano-Mislata A. Madueno F. (2007). Floral initiation and inflorescence architecture: a comparative view. Ann. Bot. 100, 659–676. 10.1093/aob/mcm146 17679690 PMC2533596

[B5] Berbel A. Ferrándiz C. Hecht V. Dalmais M. Lund O. S. Sussmilch F. C.. (2012). VEGETATIVE1 is essential for development of the compound inflorescence in pea. Nat. Commun. 3:797. 10.1038/ncomms1801 22531182

[B6] Bernardo K. A. S. Freire Filho F. R. Ribeiro V. Q. Vieira P. F. M. J. Lopes Â. C. A. Oliveira R. M. (2018). Incorporation of compound inflorescences and selection of high-yielding progenies in cowpea. Pesqui. Agropecuária Bras. 53, 1150–1157. 10.1590/s0100-204x2018001000008

[B7] Bradley D. Ratcliffe O. Vincent C. Carpenter R. Coen E. (1997). Inflorescence commitment and architecture in arabidopsis. Science 275, 80–83. 10.1126/science.275.5296.80 8974397

[B8] Chankaew S. Somta P. Isemura T. Tomooka N. Kaga A. Vaughan D. A.. (2013). Quantitative trait locus mapping reveals conservation of major and minor loci for powdery mildew resistance in four sources of resistance in mungbean [*Vigna radiata* (L.) Wilczek]. Mol. Breed. 32, 121–130. 10.1007/s11032-013-9856-6

[B9] Chankaew S. Somta P. Sorajjapinun W. Srinives P. (2011). Quantitative trait loci mapping of cercospora leaf spot resistance in mungbean, *Vigna radiata* (L.) Wilczek. Mol. Breed. 28, 255–264. 10.1007/s11032-010-9478-1

[B10] Chen H.-M. Ku H.-M. Schafleitner R. Bains T. S. George Kuo C. Liu C.-A.. (2013). The major quantitative trait locus for mungbean yellow mosaic Indian virus resistance is tightly linked in repulsion phase to the major bruchid resistance locus in a cross between mungbean [*Vigna radiata* (L.) wilczek] and its wild relative *Vigna radiata* ssp. sublobata. Euphytica 192, 205–216. 10.1007/s10681-012-0831-9

[B11] Chotechung S. Somta P. Chen J. Yimram T. Chen X. Srinives P. (2016). A gene encoding a polygalacturonase-inhibiting protein (PGIP) is a candidate gene for bruchid (*Coleoptera: Bruchidae*) resistance in mungbean (*Vigna radiata*). Theor. Appl. Genet. 129, 1673–1683. 10.1007/s00122-016-2731-1 27220975

[B12] Danecek P. Auton A. Abecasis G. Albers C. A. Banks E. DePristo M. A.. (2011). The variant call format and VCFtools. Bioinformatics 27, 2156–2158. 10.1093/bioinformatics/btr330 21653522 PMC3137218

[B13] Dong Y. Yang X. Liu J. Wang B.-H. Liu B.-L. Wang Y.-Z. (2014). Pod shattering resistance associated with domestication is mediated by a NAC gene in soybean. Nat. Commun. 5:3352. 10.1038/ncomms4352 24549030

[B14] Elshire R. J. Glaubitz J. C. Sun Q. Poland J. A. Kawamoto K. Buckler E. S.. (2011). A robust, simple genotyping-by-sequencing (GBS) approach for high diversity species. PLoS ONE 6:e19379. 10.1371/journal.pone.0019379 21573248 PMC3087801

[B15] Han Y. Yang H. Jiao Y. (2014). Regulation of inflorescence architecture by cytokinins. Front. Plant Sci. 5:669. 10.3389/fpls.2014.00669 25505480 PMC4241816

[B16] Hwang W. J. Ha J. Lee T. Jeong H. Kim M. Y. Kim S. K.. (2017). A candidate flowering gene in mungbean is homologous to a soybean phytochrome A gene. Euphytica 213:79. 10.1007/s10681-017-1866-8

[B17] IBPGR (1985). Descriptors for Vigna mungo and V. radiata (revised). Rome: IBPGR Secretariat.

[B18] Isemura T. Kaga A. Tabata S. Somta P. Srinives P. Shimizu T.. (2012). Construction of a genetic linkage map and genetic analysis of domestication related traits in mungbean (*Vigna radiata*). PLoS ONE 7:e41304. 10.1371/journal.pone.0041304 22876284 PMC3410902

[B19] Jin J. Tian F. Yang D.-C. Meng Y.-Q. Kong L. Luo J.. (2017). PlantTFDB 4.0: toward a central hub for transcription factors and regulatory interactions in plants. Nucleic Acids Res. 45, D1040–D1045. 10.1093/nar/gkw982 27924042 PMC5210657

[B20] Kaewwongwal A. Chen J. Somta P. Kongjaimun A. Yimram T. Chen X.. (2017). Novel alleles of two tightly linked genes encoding polygalacturonase-inhibiting proteins (VrPGIP1 and VrPGIP2) associated with the Br locus that confer bruchid (*Callosobruchus* spp.) resistance to mungbean (*Vigna radiata*) accession V2709. Front. Plant Sci. 8:1692. 10.3389/fpls.2017.01692 29033965 PMC5625325

[B21] Kaewwongwal A. Liu C. Somta P. Chen J. Tian J. Yuan X.. (2020). A second VrPGIP1 allele is associated with bruchid resistance (*Callosobruchus* spp.) in wild mungbean (*Vigna radiata* var. sublobata) accession ACC41. Mol. Genet. Genomics 295, 275–286. 10.1007/s00438-019-01619-y 31705195

[B22] Kang Y. J. Kim S. K. Kim M. Y. Lestari P. Kim K. H. Ha B.-K.. (2014). Genome sequence of mungbean and insights into evolution within Vigna species. Nat. Commun. 5:5443. 10.1038/ncomms6443 25384727 PMC4241982

[B23] Kim K.-S. Diers B. W. Hyten D. L. Mian M. R. Shannon J. G. Nelson R. L. (2012). Identification of positive yield QTL alleles from exotic soybean germplasm in two backcross populations. Theor. Appl. Genet. 125, 1353–1369. 10.1007/s00122-012-1944-1 22869284

[B24] Kim S. K. Nair R. M. Lee J. Lee S.-H. (2015). Genomic resources in mungbean for future breeding programs. Front. Plant Sci. 6:626. 10.3389/fpls.2015.00626 26322067 PMC4530597

[B25] Kitsanachandee R. Somta P. Chatchawankanphanich O. Akhtar K. P. Shah T. M. Nair R. M.. (2013). Detection of quantitative trait loci for mungbean yellow mosaic India virus (MYMIV) resistance in mungbean (*Vigna radiata* (L.) wilczek) in India and Pakistan. Breed. Sci. 63, 367–373. 10.1270/jsbbs.63.367 24399908 PMC3859347

[B26] Kuroda Y. Kaga A. Tomooka N. Yano H. Takada Y. Kato S.. (2013). QTL affecting fitness of hybrids between wild and cultivated soybeans in experimental fields. Ecol. Evol. 3, 2150–2168. 10.1002/ece3.606 23919159 PMC3728954

[B27] Lark K. G. Chase K. Adler F. Mansur L. M. Orf J. H. (1995). Interactions between quantitative trait loci in soybean in which trait variation at one locus is conditional upon a specific allele at another. Proc. Natl. Acad. Sci. U.S.A. 92, 4656–4660. 10.1073/pnas.92.10.4656 7753859 PMC42003

[B28] Li H. (2013). Aligning sequence reads, clone sequences and assembly contigs with BWA-MEM. ArXiv13033997 Q-Bio. Available online at: http://arxiv.org/abs/1303.3997 (accessed June 8, 2020).

[B29] Li H. Handsaker B. Wysoker A. Fennell T. Ruan J. Homer N.. (2009). The sequence alignment/map format and SAMtools. Bioinformatics 25, 2078–2079. 10.1093/bioinformatics/btp352 19505943 PMC2723002

[B30] Li S. Ding Y. Zhang D. Wang X. Tang X. Dai D.. (2018). Parallel domestication with a broad mutational spectrum of determinate stem growth habit in leguminous crops. Plant J. 96, 761–771. 10.1111/tpj.14066 30112860

[B31] Liljegren S. J. Gustafson-Brown C. Pinyopich A. Ditta G. S. Yanofsky M. F. (1999). Interactions among APETALA1, LEAFY, and TERMINAL FLOWER1 specify meristem fate. Plant Cell 11, 1007–1018. 10.1105/tpc.11.6.1007 10368173 PMC144247

[B32] Liu B. Watanabe S. Uchiyama T. Kong F. Kanazawa A. Xia Z.. (2010). The soybean stem growth habit gene Dt1 is an ortholog of arabidopsis TERMINAL FLOWER1. Plant Physiol. 153, 198–210. 10.1104/pp.109.150607 20219831 PMC2862436

[B33] Livak K. J. Schmittgen T. D. (2001). Analysis of relative gene expression data using real-time quantitative PCR and the 2–ΔΔCT method. Methods 25, 402–408. 10.1006/meth.2001.1262 11846609

[B34] Mansur L. M. Orf J. H. Chase K. Jarvik T. Cregan P. B. Lark K. G. (1996). Genetic mapping of agronomic traits using recombinant inbred lines of soybean. Crop Sci. 36, 1327–1336. 10.2135/cropsci1996.0011183X003600050042x

[B35] Mathivathana M. K. Murukarthick J. Karthikeyan A. Jang W. Dhasarathan M. Jagadeeshselvam N.. (2019). Detection of QTLs associated with mungbean yellow mosaic virus (MYMV) resistance using the interspecific cross of *Vigna radiata* × *Vigna umbellata*. J. Appl. Genet. 60, 255–268. 10.1007/s13353-019-00506-x 31332718

[B36] Mehandi S. Quatadah S. Mishra S. P. Singh I. Praveen N. Dwivedi N. (2019). Mungbean (*Vigna radiata* L. wilczek): retrospect and prospects, in Legume Crops - Characterization and Breeding for Improved Food Security, ed. El-Esawi M. A. (London: IntechOpen), 49–66. 10.5772/intechopen.85657

[B37] Mei H. Liu Y. Du Z. Wu K. Cui C. Jiang X.. (2017). High-density genetic map construction and gene mapping of basal branching habit and flowers per leaf axil in sesame. Front. Plant Sci. 8:636. 10.3389/fpls.2017.00636 28496450 PMC5406510

[B38] Meng L. Li H. Zhang L. Wang J. (2015). QTL IciMapping: integrated software for genetic linkage map construction and quantitative trait locus mapping in biparental populations. Crop J. 3, 269–283. 10.1016/j.cj.2015.01.001

[B39] Orf J. H. Chase K. Jarvik T. Mansur L. M. Cregan P. B. Adler F. R.. (1999). Genetics of soybean agronomic traits: I. Comparison of three related recombinant inbred populations. Crop Sci. 39, 1642–1651. 10.2135/cropsci1999.3961642x

[B40] Pereira L. Ruggieri V. Pérez S. Alexiou K. G. Fernández M. Jahrmann T.. (2018). QTL mapping of melon fruit quality traits using a high-density GBS-based genetic map. BMC Plant Biol. 18:324. 10.1186/s12870-018-1537-5 30509167 PMC6278158

[B41] Prusinkiewicz P. Erasmus Y. Lane B. Harder L. D. Coen E. (2007). Evolution and development of inflorescence architectures. Science 316, 1452–1456. 10.1126/science.1140429 17525303

[B42] Schafleitner R. Huang S. Chu S. Yen J. Lin C. Yan M.. (2016). Identification of single nucleotide polymorphism markers associated with resistance to bruchids (*Callosobruchus* spp.) in wild mungbean (*Vigna radiata* var. sublobata) and cultivated *V. radiata* through genotyping by sequencing and quantitative trait locus analysis. BMC Plant Biol. 16:159. 10.1186/s12870-016-0847-8 27422285 PMC4946214

[B43] Selvi R. Muthiah A. R. Maheswaran M. Shanmugasundaram P. (2003). Genetic diversity analysis in the genus vigna based on morphological traits and isozyme markers. Sabrao J. Breed. Genet. 35, 103–112.

[B44] Sen N. K. Ghosh A. K. (1959). Genetic studies in green gram. Indian J. Genet. 19, 210–227.

[B45] Shanmugasundaram S. Keatinge J. D. H. Hughes J. D. (2009). Counting on beans: mungbean improvement in Asia, in Millions Fed: Proven Successes in Agricultural Development, eds Spielman D. J. Pandya-Lorch R. (Washington, DC: International Food Policy Research Institute), 103–108.

[B46] Shannon S. Meeks-Wagner D. R. (1993). Genetic interactions that regulate inflorescence development in arabidopsis. Plant Cell 5, 639–655. 10.2307/3869807 12271079 PMC160302

[B47] Shao J. Liu X. Wang R. Zhang G. Yu F. (2012). The over-expression of an arabidopsis B3 transcription factor, ABS2/NGAL1, leads to the loss of flower petals. PLoS ONE 7:e49861. 10.1371/journal.pone.0049861 23185464 PMC3503873

[B48] Shim S. Kim M. Y. Ha J. Lee Y.-H. Lee S.-H. (2017). Identification of QTLs for branching in soybean (*Glycine max* (L.) merrill). Euphytica 213, 225. 10.1007/s10681-017-2016-z30609682

[B49] Singh T. P. Singh K. B. (1970). Inheritance of clusters per node in mungbean (*Phaseolus aureus* Roxb.). Curr. Sci. 39:265.

[B50] Tian Z. Wang X. Lee R. Li Y. Specht J. E. Nelson R. L.. (2010). Artificial selection for determinate growth habit in soybean. Proc. Natl. Acad. Sci. U.S.A. 107, 8563–8568. 10.1073/pnas.1000088107 20421496 PMC2889302

[B51] Tucker S. C. (2003). Floral development in legumes. Plant Physiol. 131, 911–926. 10.1104/pp.102.017459 12644644 PMC1540291

[B52] Ungerer M. C. Halldorsdottir S. S. Modliszewski J. L. Mackay T. F. C. Purugganan M. D. (2002). Quantitative trait loci for inflorescence development in arabidopsis thaliana. Genetics 160, 1133–1151. 10.1093/genetics/160.3.1133 11901129 PMC1462026

[B53] Van Ooijen J. (2006). JoinMap^®^ 4, Software for the Calculation of Genetic Linkage Maps in Experimental Populations. Kyazma BV Wagening.

[B54] Wang R. Liu L. Kong J. Xu Z. Akhter Bhat J. Zhao T. (2019). QTL architecture of vine growth habit and gibberellin oxidase gene diversity in wild soybean (*Glycine soja*). Sci. Rep. 9:7393. 10.1038/s41598-019-43887-z 31089185 PMC6517428

[B55] Wang S. Guo T. Wang Z. Kang J. Yang Q. Shen Y.. (2020). Expression of three related to ABI3/VP1 genes in medicago truncatula caused increased stress resistance and branch increase in arabidopsis thaliana. Front. Plant Sci. 11:611. 10.3389/fpls.2020.00611 32523590 PMC7261895

[B56] Xia Z. Watanabe S. Yamada T. Tsubokura Y. Nakashima H. Zhai H.. (2012). Positional cloning and characterization reveal the molecular basis for soybean maturity locus E1 that regulates photoperiodic flowering. Proc. Natl. Acad. Sci. U.S.A. 109, E2155–E2164. 10.1073/pnas.1117982109 22619331 PMC3420212

[B57] Yoon M. Y. Kim M. Y. Ha J. Lee T. Kim K. D. Lee S.-H. (2019). QTL analysis of resistance to high-intensity UV-B irradiation in soybean (*Glycine max* [L.] Merr.). Int. J. Mol. Sci. 20:3287. 10.3390/ijms20133287 31277435 PMC6651677

[B58] Yundaeng C. Somta P. Chen J. Yuan X. Chankaew S. Chen X. (2021). Fine mapping of QTL conferring Cercospora leaf spot disease resistance in mungbean revealed TAF5 as candidate gene for the resistance. Theor. Appl. Genet. 134, 701–714. 10.1007/s00122-020-03724-8 33188437

[B59] Yundaeng C. Somta P. Chen J. Yuan X. Chankaew S. Srinives P.. (2020). Candidate gene mapping reveals VrMLO12 (MLO Clade II) is associated with powdery mildew resistance in mungbean (*Vigna radiata* [L.] wilczek). Plant Sci. 298:110594. 10.1016/j.plantsci.2020.110594 32771151

[B60] Zhang D. Cheng H. Wang H. Zhang H. Liu C. Yu D. (2010). Identification of genomic regions determining flower and pod numbers development in soybean (*Glycine max* L.). J. Genet. Genomics 37, 545–556. 10.1016/S1673-8527(09)60074-6 20816387

