# Supplementary material for: Identification of a Locus Controlling Compound Raceme Inflorescence in Mungbean [Vigna radiata (L.) R. Wilczek]
Source: Front Genet. 2021 Mar 8;12:642518. doi: 10.3389/fgene.2021.642518 (PMC7982598; doi:10.3389/fgene.2021.642518)
Supplement: Supplementary file 1 [file Data_Sheet_1.docx]

Supplementary Material

# Supplementary Data

**Supplementary Tables**

**Supplementary Table 1.** Barcode adapter sequences used for GBS library construction.

| Barcode | adapter_top | adapter_bottom |
| --- | --- | --- |
| TGACGCCA | cacgacgctcttccgatctTGACGCCA | CWGTGGCGTCAagatcggaagagcgtcgtg |
| TGCCGCAT | cacgacgctcttccgatctTGCCGCAT | CWGATGCGGCAagatcggaagagcgtcgtg |
| TATTCGCAT | cacgacgctcttccgatctTATTCGCAT | CWGATGCGAATAagatcggaagagcgtcgtg |
| ATAGAT | cacgacgctcttccgatctATAGAT | CWGATCTATagatcggaagagcgtcgtg |
| GGTGT | cacgacgctcttccgatctGGTGT | CWGACACCagatcggaagagcgtcgtg |
| GGATA | cacgacgctcttccgatctGGATA | CWGTATCCagatcggaagagcgtcgtg |
| TATCGGGA | cacgacgctcttccgatctTATCGGGA | CWGTCCCGATAagatcggaagagcgtcgtg |
| CGCTT | cacgacgctcttccgatctCGCTT | CWGAAGCGagatcggaagagcgtcgtg |
| AACGCACATT | cacgacgctcttccgatctAACGCACATT | CWGAATGTGCGTTagatcggaagagcgtcgtg |
| TAGCGGAT | cacgacgctcttccgatctTAGCGGAT | CWGATCCGCTAagatcggaagagcgtcgtg |
| GGTATA | cacgacgctcttccgatctGGTATA | CWGTATACCagatcggaagagcgtcgtg |
| TCTTGG | cacgacgctcttccgatctTCTTGG | CWGCCAAGAagatcggaagagcgtcgtg |
| TGGCAACAGA | cacgacgctcttccgatctTGGCAACAGA | CWGTCTGTTGCCAagatcggaagagcgtcgtg |
| GCGT | acactctttccctacacgacgctcttccgatctGCGT | CWGACGCagatcggaagagcgtcgtgtagggaaagagtgt |
| TAGCATGC | cacgacgctcttccgatctTAGCATGC | CWGGCATGCTAagatcggaagagcgtcgtg |
| ATATGT | cacgacgctcttccgatctATATGT | CWGACATATagatcggaagagcgtcgtg |
| CACCA | cacgacgctcttccgatctCACCA | CWGTGGTGagatcggaagagcgtcgtg |
| CCTTGCCATT | cacgacgctcttccgatctCCTTGCCATT | CWGAATGGCAAGGagatcggaagagcgtcgtg |
| TTCGTT | cacgacgctcttccgatctTTCGTT | CWGAACGAAagatcggaagagcgtcgtg |
| ATATAA | cacgacgctcttccgatctATATAA | CWGTTATATagatcggaagagcgtcgtg |
| CGTGGACAGT | cacgacgctcttccgatctCGTGGACAGT | CWGACTGTCCACGagatcggaagagcgtcgtg |
| TGGCACAGA | cacgacgctcttccgatctTGGCACAGA | CWGTCTGTGCCAagatcggaagagcgtcgtg |
| GTCGATT | cacgacgctcttccgatctGTCGATT | CWGAATCGACagatcggaagagcgtcgtg |
| ATCGTA | cacgacgctcttccgatctATCGTA | CWGTACGATagatcggaagagcgtcgtg |
| TGCA | acactctttccctacacgacgctcttccgatctTGCA | CWGTGCAagatcggaagagcgtcgtgtagggaaagagtgt |
| ACTGCGAT | cacgacgctcttccgatctACTGCGAT | CWGATCGCAGTagatcggaagagcgtcgtg |
| ACAACT | cacgacgctcttccgatctACAACT | CWGAGTTGTagatcggaagagcgtcgtg |
| ACTGCT | cacgacgctcttccgatctACTGCT | CWGAGCAGTagatcggaagagcgtcgtg |
| CTTGA | cacgacgctcttccgatctCTTGA | CWGTCAAGagatcggaagagcgtcgtg |
| GCGTCCT | cacgacgctcttccgatctGCGTCCT | CWGAGGACGCagatcggaagagcgtcgtg |
| CATCGT | cacgacgctcttccgatctCATCGT | CWGACGATGagatcggaagagcgtcgtg |
| TAATA | cacgacgctcttccgatctTAATA | CWGTATTAagatcggaagagcgtcgtg |
| CTCC | acactctttccctacacgacgctcttccgatctCTCC | CWGGGAGagatcggaagagcgtcgtgtagggaaagagtgt |
| GGAACGA | cacgacgctcttccgatctGGAACGA | CWGTCGTTCCagatcggaagagcgtcgtg |
| AACTGG | cacgacgctcttccgatctAACTGG | CWGCCAGTTagatcggaagagcgtcgtg |
| ATGAGCAA | cacgacgctcttccgatctATGAGCAA | CWGTTGCTCATagatcggaagagcgtcgtg |
| CTCTA | cacgacgctcttccgatctCTCTA | CWGTAGAGagatcggaagagcgtcgtg |
| GGTGCACATT | cacgacgctcttccgatctGGTGCACATT | CWGAATGTGCACCagatcggaagagcgtcgtg |
| ACGACTAC | cacgacgctcttccgatctACGACTAC | CWGGTAGTCGTagatcggaagagcgtcgtg |
| ATTAATT | cacgacgctcttccgatctATTAATT | CWGAATTAATagatcggaagagcgtcgtg |
| GAGCGACAT | cacgacgctcttccgatctGAGCGACAT | CWGATGTCGCTCagatcggaagagcgtcgtg |
| GAACTTC | cacgacgctcttccgatctGAACTTC | CWGGAAGTTCagatcggaagagcgtcgtg |
| TAGCCAA | cacgacgctcttccgatctTAGCCAA | CWGTTGGCTAagatcggaagagcgtcgtg |
| ATATCGCCA | cacgacgctcttccgatctATATCGCCA | CWGTGGCGATATagatcggaagagcgtcgtg |
| GGCTTA | cacgacgctcttccgatctGGCTTA | CWGTAAGCCagatcggaagagcgtcgtg |
| CGTCGCCACT | cacgacgctcttccgatctCGTCGCCACT | CWGAGTGGCGACGagatcggaagagcgtcgtg |
| GTGAGGGT | cacgacgctcttccgatctGTGAGGGT | CWGACCCTCACagatcggaagagcgtcgtg |
| GTACTT | cacgacgctcttccgatctGTACTT | CWGAAGTACagatcggaagagcgtcgtg |
| GCGCTCA | cacgacgctcttccgatctGCGCTCA | CWGTGAGCGCagatcggaagagcgtcgtg |
| TATCA | cacgacgctcttccgatctTATCA | CWGTGATAagatcggaagagcgtcgtg |
| GTCGCCT | cacgacgctcttccgatctGTCGCCT | CWGAGGCGACagatcggaagagcgtcgtg |
| AATAACCAA | cacgacgctcttccgatctAATAACCAA | CWGTTGGTTATTagatcggaagagcgtcgtg |
| AACT | cacgacgctcttccgatctACAACT | CWGAGTTGTagatcggaagagcgtcgtg |
| CAAGT | cacgacgctcttccgatctCAAGT | CWGACTTGagatcggaagagcgtcgtg |
| TTCCTGGA | cacgacgctcttccgatctTTCCTGGA | CWGTCCAGGAAagatcggaagagcgtcgtg |
| TAGGAA | cacgacgctcttccgatctTAGGAA | CWGTTCCTAagatcggaagagcgtcgtg |
| GCTGTGGA | cacgacgctcttccgatctGCTGTGGA | CWGTCCACAGCagatcggaagagcgtcgtg |
| CAGA | acactctttccctacacgacgctcttccgatctCAGA | CWGTCTGagatcggaagagcgtcgtgtagggaaagagtgt |
| CTCAT | cacgacgctcttccgatctCTCAT | CWGATGAGagatcggaagagcgtcgtg |
| ACGGTACT | cacgacgctcttccgatctACGGTACT | CWGAGTACCGTagatcggaagagcgtcgtg |
| CGTGTGGT | cacgacgctcttccgatctCGTGTGGT | CWGACCACACGagatcggaagagcgtcgtg |
| ACAGT | cacgacgctcttccgatctACAGT | CWGACTGTagatcggaagagcgtcgtg |
| GGATTGGT | cacgacgctcttccgatctGGATTGGT | CWGACCAATCCagatcggaagagcgtcgtg |
| AGGAT | cacgacgctcttccgatctAGGAT | CWGATCCTagatcggaagagcgtcgtg |
| GCGTACAAT | cacgacgctcttccgatctGCGTACAAT | CWGATTGTACGCagatcggaagagcgtcgtg |
| TCAC | acactctttccctacacgacgctcttccgatctTCAC | CWGGTGAagatcggaagagcgtcgtgtagggaaagagtgt |
| TTGCTG | cacgacgctcttccgatctTTGCTG | CWGCAGCAAagatcggaagagcgtcgtg |
| CAGTGCCATT | cacgacgctcttccgatctCAGTGCCATT | CWGAATGGCACTGagatcggaagagcgtcgtg |
| ACAACCAACT | cacgacgctcttccgatctACAACCAACT | CWGAGTTGGTTGTagatcggaagagcgtcgtg |
| TGCAGA | cacgacgctcttccgatctTGCAGA | CWGTCTGCAagatcggaagagcgtcgtg |
| TTCTC | cacgacgctcttccgatctTTCTC | CWGGAGAAagatcggaagagcgtcgtg |
| ACCTAA | cacgacgctcttccgatctACCTAA | CWGTTAGGTagatcggaagagcgtcgtg |
| AACGTGCCT | cacgacgctcttccgatctAACGTGCCT | CWGAGGCACGTTagatcggaagagcgtcgtg |
| GCACGAT | cacgacgctcttccgatctGCACGAT | CWGATCGTGCagatcggaagagcgtcgtg |
| TATGT | cacgacgctcttccgatctTATGT | CWGACATAagatcggaagagcgtcgtg |
| TGCGA | acactctttccctacacgacgctcttccgatctTGCGA | CWGTCGCAagatcggaagagcgtcgtgtagggaaagagtgt |
| GTAA | acactctttccctacacgacgctcttccgatctGTAA | CWGTTACagatcggaagagcgtcgtgtagggaaagagtgt |
| CGTTCA | cacgacgctcttccgatctCGTTCA | CWGTGAACGagatcggaagagcgtcgtg |
| AATATGC | cacgacgctcttccgatctAATATGC | CWGGCATATTagatcggaagagcgtcgtg |
| ATGAAAC | cacgacgctcttccgatctATGAAAC | CWGGTTTCATagatcggaagagcgtcgtg |
| GGACCTA | cacgacgctcttccgatctGGACCTA | CWGTAGGTCCagatcggaagagcgtcgtg |
| GTGACACAT | cacgacgctcttccgatctGTGACACAT | CWGATGTGTCACagatcggaagagcgtcgtg |
| GAATGCAATA | cacgacgctcttccgatctGAATGCAATA | CWGTATTGCATTCagatcggaagagcgtcgtg |
| GCTCCGA | cacgacgctcttccgatctGCTCCGA | CWGTCGGAGCagatcggaagagcgtcgtg |
| TCAGAGAT | cacgacgctcttccgatctTCAGAGAT | CWGATCTCTGAagatcggaagagcgtcgtg |
| TAGGCCAT | cacgacgctcttccgatctTAGGCCAT | CWGATGGCCTAagatcggaagagcgtcgtg |
| TGCAAGGA | cacgacgctcttccgatctTGCAAGGA | CWGTCCTTGCAagatcggaagagcgtcgtg |
| GAATTCA | cacgacgctcttccgatctGAATTCA | CWGTGAATTCagatcggaagagcgtcgtg |
| CATAT | cacgacgctcttccgatctCATAT | CWGATATGagatcggaagagcgtcgtg |
| AAGACGCT | cacgacgctcttccgatctAAGACGCT | CWGAGCGTCTTagatcggaagagcgtcgtg |
| CAACCACACA | cacgacgctcttccgatctCAACCACACA | CWGTGTGTGGTTGagatcggaagagcgtcgtg |
| CCGAACA | cacgacgctcttccgatctCCGAACA | CWGTGTTCGGagatcggaagagcgtcgtg |
| GGAAGACAT | cacgacgctcttccgatctGGAAGACAT | CWGATGTCTTCCagatcggaagagcgtcgtg |
| AACGCCT | cacgacgctcttccgatctAACGCCT | CWGAGGCGTTagatcggaagagcgtcgtg |
| CTGTA | cacgacgctcttccgatctCTGTA | CWGTACAGagatcggaagagcgtcgtg |
| CCGGATAT | cacgacgctcttccgatctCCGGATAT | CWGATATCCGGagatcggaagagcgtcgtg |

**Supplementary Table 2.** List of primers used for validation of sequence variation and qRT-PCR analysis of selected genes.

| Purpose | Gene ID | Primer sequence (5ʹ-3ʹ) | PCR product size |
| --- | --- | --- | --- |
| Sanger sequencing | *Vradi04g00002442*-1 | F: AGACCAAGCAGAGCTAAGAGG  R: CCAAGGTTGAGATTGAGGGTGA | 1134 |
|  | *Vradi04g00002442*-2 | F: GAGAAACGGTGGCTGGCTA  R: GTCCACACACGAACAAGATGT | 1200 |
|  | *Vradi04g00002481*-1 | F: TCAACGCTTTGTTACTTCTAACACT  R: ACAACTCTGCGATCTCACTTTCA | 1195 |
|  | *Vradi04g00002481*-2 | F: TTTGATGTGGAAGCTGGGGG  R: TGAGTAGACAGCAATAGGACCA | 1168 |
| qRT-PCR | *Vradi04g00002442* | F: GCCATGAGTTTTTCCCTTCA  R: CCTGGAACATCAGGGTCTGT |  |
|  | *Vradi04g00002481* | F: CTCCATTCTTGTCCCGAAGA  R: AACCCCAAACAAGGAAAAGG |  |
|  | *Vradi05g00001056* (*VreIF-5a*) | F: ACCTTAGGCTTCCAACCGAT  R: CTCCCATTGCAGACATAACAGA |  |

F: forward; R: reverse.

**Supplementary Table 3.** Segregation analysis of compound and simple raceme in the RIL population derived from VC1973A × IT208075.

| Parent or progeny | No. of plants/lines | | | Total | Expected ratio | *X*^2^ | *P-value* |
| --- | --- | --- | --- | --- | --- | --- | --- |
|  | Compound | Segregation | Simple |  |  |  |  |
| VC1973A | 20 | 0 | 0 | 20 |  |  |  |
| IT208075 | 0 | 0 | 20 | 20 |  |  |  |
| F_8:9_ | 122 | 14 | 99 | 235 | 1:1 | 2.4 | 0.12 ns |

ns: not significant.

**Supplementary Table 4.** Statistic summary of resequencing of IT208075, GBS of the RIL population, and linkage map construction.

| Purpose | Category | IT208075 (resequencing) |  | RILs (GBS) |
| --- | --- | --- | --- | --- |
| Sequencing | Total bases | 4.3 Gb (9×) |  |  |
|  | Total reads | 31,033,916 |  |  |
|  | Mapped bases | 3,343,405,181 |  |  |
|  | Mapped reads | 23,155,680 |  | 672,028,074 |
|  | Mapped reads per line |  |  | 2,680,760  (1,202,609–6,565,325) |
|  | Coverage (%) | 91.2% |  | 2.2% (1.3–3.4%) |
|  | Mapping depth (×) | 7.7× |  | 13.9× (5.6–27.1×) |
|  | Category | | | No. of markers |
| Map construction | Total SNPs | | | 2,351,549 |
|  | Total SNPs after filtering | | | 10,989 |
|  | No. of SNPs used in map construction | | | 4,177 |
|  | No. of SNPs incorporated in the linkage map | | | 1,799 |

GBS: genotyping-by-sequencing.

Mapped reads per line, coverage, and mapping depth of RILs represent means; values inside the parentheses represent the range of population.

**Supplementary Table 5.** Number of SNP markers, genetic distance, and average marker distance by chromosome in the genetic linkage map of the VC1973A × IT208075 RIL population.

| Chromosome | No. of SNP markers | Linkage distance (cM) | | Average marker distance (cM) | |
| --- | --- | --- | --- | --- | --- |
|  |  | ML | Reg. | ML | Reg. |
| Vr01 | 333 | 195.3 | 43.4 | 0.59 | 0.13 |
| Vr02 | 357 | 261.1 | 56.1 | 0.73 | 0.16 |
| Vr03 | 208 | 186.2 | 59.6 | 0.90 | 0.29 |
| Vr04 | 160 | 90.8 | 29.8 | 0.57 | 0.19 |
| Vr05 | 119 | 81.3 | 27.3 | 0.68 | 0.23 |
| Vr06 | 166 | 128.0 | 56.0 | 0.77 | 0.34 |
| Vr07 | 54 | 42.2 | 19.9 | 0.78 | 0.37 |
| Vr08 | 76 | 106.3 | 54.9 | 1.40 | 0.72 |
| Vr09 | 159 | 140.9 | 52.6 | 0.89 | 0.33 |
| Vr10 | 110 | 44.6 | 6.2 | 0.41 | 0.06 |
| Vr11 | 57 | 76.8 | 44.2 | 1.35 | 0.77 |
| Average | 163.5 | 123.0 | 40.9 | 0.82 | 0.33 |
| Total | 1799 | 1353.5 | 450.1 |  |  |

ML: maximum likely hood mapping method; Reg: regression mapping method.

**Supplementary Table 7.** Lists of 64 genes located in the *Comraceme* locus, their sequence variation between VC1973A and IT208075, and annotated functions based on *Arabidopsis thaliana* homologous genes.

| *V.radiata* gene ID | 2 kb upstream | 5’UTR | CDS | | | Intron | 3’UTR | 2 kb downstream | *A. thaliana* gene ID | Gene function^a^ |
| --- | --- | --- | --- | --- | --- | --- | --- | --- | --- | --- |
|  |  |  | Synonymous | Missense | Frameshift |  |  |  |  |  |
| *Vradi04g00002436* | 1 | 0 | 1 | 3 | 0 | 5 | 1 | 1 | *AT5G03610* | GDSL-like Lipase/Acylhydrolase superfamily protein |
| *Vradi04g00002437* | 4 | 1 | 0 | 0 | 0 | 1 | 0 | 4 | *AT2G36310* | uridine-ribohydrolase 1 |
| *Vradi04g00002438* | 1 | 0 | 0 | 0 | 0 | 0 | 0 | 1 | *AT2G36320* | A20/AN1-like zinc finger family protein |
| *Vradi04g00002439* | 1 | 1 | 0 | 0 | 0 | 0 | 0 | 1 | *AT3G52820* | purple acid phosphatase 22 |
| *Vradi04g00002440* | 0 | 0 | 0 | 0 | 0 | 1 | 0 | 0 | *AT3G52780* | Purple acid phosphatases superfamily protein |
| *Vradi04g00002441* | 6 | 0 | 0 | 0 | 0 | 0 | 0 | 6 | *AT3G52760* | Integral membrane Yip1 family protein |
| *Vradi04g00002442* | 2 | 0 | 0 | 0 | 0 | 2 | 0 | 2 | *AT2G27550* | centroradialis (PEBP) |
| *Vradi04g00002443* | 1 | 1 | 0 | 0 | 0 | 11 | 0 | 1 | *AT2G36290* | alpha/beta-Hydrolases superfamily protein |
| *Vradi04g00002444* | 0 | 0 | 0 | 0 | 0 | 0 | 0 | 0 | *AT1G67190* | F-box/RNI-like superfamily protein |
| *Vradi04g00002445* | 4 | 0 | 0 | 0 | 1 | 14 | 2 | 4 | *AT2G16710* | Iron-sulphur cluster biosynthesis family protein |
| *Vradi04g00002446* | 10 | 2 | 0 | 0 | 0 | 6 | 0 | 10 | *AT2G36250* | Tubulin/FtsZ family protein |
| *Vradi04g00002447* | 5 | 0 | 0 | 0 | 0 | 0 | 0 | 5 | *AT5G15770* | glucose-6-phosphate acetyltransferase 1 |
| *Vradi04g00002448* | 0 | 0 | 0 | 0 | 0 | 0 | 0 | 0 |  |  |
| *Vradi04g00002449* | 7 | 0 | 0 | 0 | 2 | 1 | 0 | 7 | *AT2G26060* | Transducin/WD40 repeat-like superfamily protein |
| *Vradi04g00002450* | 1 | 0 | 0 | 0 | 2 | 0 | 1 | 1 |  |  |
| *Vradi04g00002451* | 0 | 1 | 0 | 0 | 0 | 3 | 0 | 0 | *AT3G52730* | ubiquinol-cytochrome C reductase UQCRX/QCR9-like family protein |
| *Vradi04g00002452* | 1 | 0 | 0 | 0 | 0 | 0 | 0 | 1 | *AT2G36240* | pentatricopeptide (PPR) repeat-containing protein |
| *Vradi04g00002453* | 0 | 0 | 0 | 0 | 0 | 1 | 0 | 0 | *AT3G10130* | SOUL heme-binding family protein |
| *Vradi04g00002454* | 3 | 0 | 0 | 0 | 0 | 0 | 0 | 3 |  |  |
| *Vradi04g00002455* | 3 | 0 | 0 | 0 | 0 | 0 | 0 | 3 |  |  |
| *Vradi04g00002456* | 5 | 0 | 0 | 0 | 1 | 8 | 0 | 5 | *AT1G57790* | F-box family protein |
| *Vradi04g00002457* | 2 | 0 | 0 | 0 | 0 | 7 | 0 | 2 | *AT3G52660* | RNA-binding (RRM/RBD/RNP motifs) family protein |
| *Vradi04g00002458* | 4 | 0 | 0 | 0 | 0 | 1 | 0 | 4 | *AT4G18590* | Nucleic acid-binding, OB-fold-like protein |
| *Vradi04g00002459* | 0 | 0 | 0 | 0 | 0 | 7 | 0 | 0 |  |  |
| *Vradi04g00002460* | 0 | 0 | 0 | 0 | 0 | 1 | 0 | 0 | *AT5G03940* | chloroplast signal recognition particle 54 kDa subunit |
| *Vradi04g00002461* | 3 | 1 | 0 | 0 | 1 | 5 | 0 | 3 | *AT5G22400* | Rho GTPase activating protein with PAK-box/P21-Rho-binding domain |
| *Vradi04g00002462* | 4 | 0 | 0 | 0 | 1 | 1 | 0 | 4 |  |  |
| *Vradi04g00002463* | 1 | 0 | 0 | 0 | 0 | 0 | 0 | 1 | *AT2G23930* | probable small nuclear ribonucleoprotein G |
| *Vradi04g00002464* | 2 | 0 | 0 | 0 | 0 | 0 | 0 | 2 | *AT2G36210* | SAUR-like auxin-responsive protein family |
| *Vradi04g00002465* | 2 | 0 | 0 | 1 | 1 | 2 | 0 | 2 | *AT2G36200* | P-loop containing nucleoside triphosphate hydrolases superfamily protein |
| *Vradi04g00002466* | 4 | 0 | 0 | 0 | 1 | 3 | 0 | 4 | *AT2G36190* | cell wall invertase 4 |
| *Vradi04g00002467* | 2 | 0 | 0 | 0 | 0 | 9 | 0 | 2 | *AT4G08850* | Leucine-rich repeat receptor-like protein kinase family protein |
| *Vradi04g00002468* | 1 | 0 | 0 | 0 | 0 | 2 | 0 | 1 | *AT3G52570* | alpha/beta-Hydrolases superfamily protein |
| *Vradi04g00002469* | 2 | 0 | 0 | 0 | 0 | 0 | 0 | 2 | *AT3G15040* | Protein of unknown function, DUF584 |
| *Vradi04g00002470* | 1 | 0 | 0 | 0 | 0 | 0 | 0 | 1 | *AT3G44350* | NAC domain containing protein 61 |
| *Vradi04g00002471* | 0 | 0 | 0 | 0 | 2 | 0 | 1 | 0 | *AT3G11530* | Vacuolar protein sorting 55 (VPS55) family protein |
| *Vradi04g00002472* | 4 | 0 | 0 | 0 | 0 | 0 | 0 | 4 | *AT3G26640* | Transducin/WD40 repeat-like superfamily protein |
| *Vradi04g00002473* | 4 | 0 | 0 | 0 | 1 | 18 | 1 | 4 | *AT3G11540* | Tetratricopeptide repeat (TPR)-like superfamily protein |
| *Vradi04g00002474* | 2 | 0 | 0 | 0 | 0 | 14 | 0 | 2 | *AT2G36130* | Cyclophilin-like peptidyl-prolyl cis-trans isomerase family protein |
| *Vradi04g00002475* | 1 | 1 | 0 | 0 | 0 | 1 | 0 | 1 | *AT5G06180* | Protein of unknown function (DUF1022) |
| *Vradi04g00002476* | 3 | 1 | 0 | 0 | 1 | 7 | 0 | 3 | *AT1G08370* | decapping 1 |
| *Vradi04g00002477* | 1 | 0 | 0 | 0 | 0 | 0 | 0 | 1 | *AT5G06210* | RNA binding (RRM/RBD/RNP motifs) family protein |
| *Vradi04g00002478* | 4 | 0 | 0 | 0 | 1 | 0 | 0 | 4 | *AT2G36090* | F-box family protein |
| *Vradi04g00002479* | 3 | 0 | 0 | 0 | 0 | 7 | 0 | 3 | *AT5G06240* | embryo defective 2735 |
| *Vradi04g00002480* | 3 | 0 | 0 | 0 | 0 | 0 | 0 | 3 | *AT2G30920* | coenzyme Q 3 |
| *Vradi04g00002481* | 4 | 0 | 0 | 0 | 1 | 1 | 0 | 4 | *AT2G36080* | B3-like transcriptional factor family protein |
| *Vradi04g00002482* | 2 | 0 | 0 | 0 | 0 | 2 | 1 | 2 | *AT2G24280* | alpha/beta-Hydrolases superfamily protein |
| *Vradi04g00002483* | 0 | 1 | 0 | 0 | 1 | 0 | 0 | 0 |  |  |
| *Vradi04g00002484* | 9 | 1 | 0 | 0 | 0 | 1 | 0 | 9 | *AT3G51680* | NAD(P)-binding Rossmann-fold superfamily protein |
| *Vradi04g00002485* | 0 | 0 | 0 | 0 | 0 | 1 | 0 | 0 | *AT3G26760* | NAD(P)-binding Rossmann-fold superfamily protein |
| *Vradi04g00002486* | 3 | 0 | 0 | 0 | 0 | 2 | 0 | 3 | *AT5G06260* | TLD-domain containing nucleolar protein |
| *Vradi04g00002487* | 1 | 0 | 0 | 0 | 0 | 0 | 0 | 1 |  |  |
| *Vradi04g00002488* | 10 | 0 | 0 | 0 | 0 | 7 | 2 | 10 | *AT3G52560* | ubiquitin E2 variant 1D-4 |
| *Vradi04g00002489* | 5 | 0 | 0 | 0 | 0 | 1 | 0 | 5 | *AT3G44260* | Polynucleotidyl transferase, ribonuclease H-like superfamily protein |
| *Vradi04g00002490* | 1 | 0 | 0 | 0 | 1 | 0 | 0 | 1 | *AT5G04820* | ovate family protein 13 |
| *Vradi04g00002491* | 0 | 0 | 0 | 0 | 0 | 0 | 0 | 0 | *AT2G36026* | Ovate family protein |
| *Vradi04g00002492* | 0 | 0 | 0 | 0 | 0 | 1 | 0 | 0 | *AT3G11620* | alpha/beta-Hydrolases superfamily protein |
| *Vradi04g00002493* | 2 | 0 | 0 | 0 | 1 | 1 | 2 | 2 | *AT5G42560* | Abscisic acid-responsive (TB2/DP1, HVA22) family protein |
| *Vradi04g00002494* | 3 | 0 | 0 | 0 | 0 | 2 | 1 | 3 | *AT3G11630* | Thioredoxin superfamily protein |
| *Vradi04g00002495* | 3 | 2 | 0 | 0 | 0 | 2 | 0 | 3 | *AT4G28730* | Glutaredoxin family protein |
| *Vradi04g00002496* | 5 | 1 | 0 | 0 | 1 | 1 | 1 | 5 |  |  |
| *Vradi04g00002497* | 2 | 0 | 0 | 0 | 0 | 1 | 0 | 2 | *AT5G06300* | Putative lysine decarboxylase family protein |
| *Vradi04g00002498* | 7 | 0 | 0 | 0 | 1 | 0 | 0 | 7 | *AT3G52500* | Eukaryotic aspartyl protease family protein |
| *Vradi04g00002499* | 7 | 0 | 1 | 0 | 0 | 0 | 0 | 7 | *AT3G52500* | Eukaryotic aspartyl protease family protein |

^a^According to *Arabidopsis thaliana* genes based on the TAIR 10 version.

**Supplementary Table 8.** The 15 genes in *Comraceme* with shared sequence variation among three mungbean lines with a compound raceme (VC1973A (reference), IT026050 (landrace), and TC1966 (wild mung bean)) compared with line IT208075 that produces a simple raceme.

| Gene ID | *A.thaliana* homolog | Gene function | Position | Compound type | | |  | Simple type | Variant location | DNA/Protein level | Reference for molecular identity |
| --- | --- | --- | --- | --- | --- | --- | --- | --- | --- | --- | --- |
|  |  |  |  | VC1973A  (reference) | IT026050  (landrace) | TC1966  (wild) |  | IT208075  (alternative) |  |  |  |
| *Vradi04g00002440* | *AT3G52780* | Purple acid phosphatases superfamily protein | 27058984 | C | C | - |  | T | downstream | c.*2578C>T |  |
| *Vradi04g00002441* | *AT3G52760* | Integral membrane Yip1 family protein | 27063771 | G | G | - |  | T | upstream | c.-1858G>T |  |
|  |  |  | 27064046 | G | G | - |  | A | upstream | c.-1583G>A |  |
| *Vradi04g00002442* | *AT2G27550* | centroradialis (PEBP) | 27076585 | CACACACACACACACAT | CACACACACACACACAT | CACACACACACACACACATAT |  | CACACACACACACAT | intron | c.264-65_264-64insACAT, c.264-79_264-78delCA | Li et al. (2018) |
| *Vradi04g00002446* | *AT2G36250* | Tubulin/FtsZ family protein | 27117565 | GAAAAAAAAA | GAAAAAAAAAAAAAAA | GAAAAAAAAAAAAAAA |  | GAAAAAAAAAA | downstream | c.*1222_*1223insAAAAAA, c.*1222_*1223insA |  |
| *Vradi04g00002447* | *AT5G15770* | glucose-6-phosphate acetyltransferase 1 | 27117565 | GAAAAAAAAA | GAAAAAAAAAAAAAAA | GAAAAAAAAAAAAAAA |  | GAAAAAAAAAA | upstream | c.-272_-271insAAAAAA, c.-272_-271insA |  |
| *Vradi04g00002450* |  |  | 27131852 | AG | AG | - |  | G | downstream | c.*1347delA |  |
| *Vradi04g00002455* |  |  | 27166743 | A | A | A |  | G | upstream | c.-127A>G |  |
| *Vradi04g00002457* | *AT3G52660* | RNA-binding (RRM/RBD/RNP motifs) family protein | 27185227 | G | G | G |  | A | intron | c.480+1156G>A |  |
| *Vradi04g00002464* | *AT2G36210* | SAUR-like auxin-responsive protein family | 27224490 | G | G | G |  | T | upstream | c.-546G>T |  |
| *Vradi04g00002465* | *AT2G36200* | P-loop containing nucleoside triphosphate hydrolases superfamily protein | 27237963 | T | T | T |  | C | missense | c.2726T>C / p.Leu909Ser |  |
| *Vradi04g00002467* | *AT4G08850* | Leucine-rich repeat receptor-like protein kinase family protein | 27252668 | C | C | C |  | T | intron | c.543-736C>T |  |
|  |  |  | 27253553 | T | T | - |  | C | intron | c.543-1621T>C |  |
|  |  |  | 27255501 | C | C | C |  | T | intron | c.542+454C>T |  |
| *Vradi04g00002470* | *AT3G44350* | NAC domain containing protein 61 | 27289701 | CTATATATATATA | CTATATATATATA | CTATATATATATA |  | CTATATATATATATA | upstream | c.-124_-123insTA |  |
| *Vradi04g00002474* | *AT2G36130* | Cyclophilin-like peptidyl-prolyl cis-trans isomerase family protein | 27339883 | C | C | C |  | T | intron | c.364+1153C>T |  |
| *Vradi04g00002479* | *AT5G06240* | embryo defective 2735 | 27373584 | G | G | G |  | A | upstream | c.-5383G>A |  |
| *Vradi04g00002481* | *AT2G36080* | B3-like transcriptional factor family protein | 27387367 | TCTCTCTCTCTCTCTCTCA | TCTCTCTCTCTCTCTCTCA | TCTCTCTCTCTCTCTCTCA |  | TCTCTCTCTCTCTCTCTCTCA | upstream | c.-566_-565insTC | Shao et al. (2012) |

**Supplementary Figures**


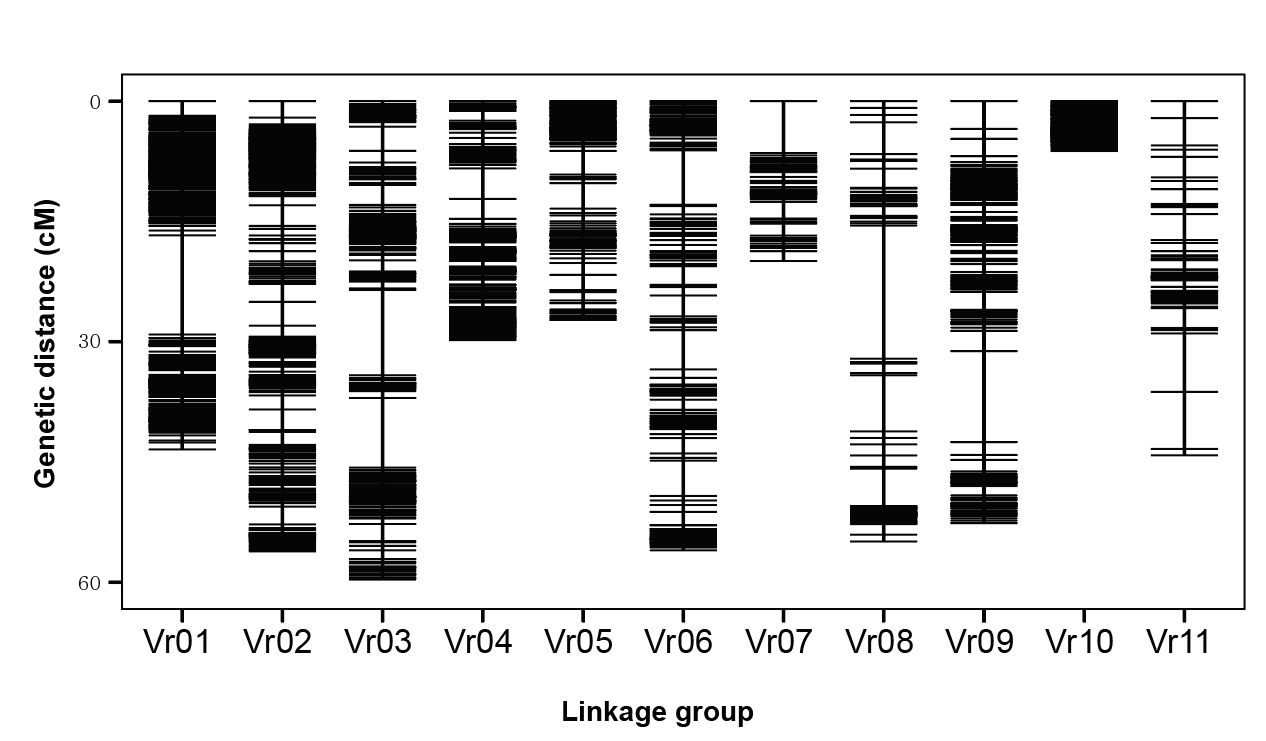


**Supplementary Figure 1.** Genetic linkage map of the VC1973A × IT208075 RIL population constructed using the regression mapping method.


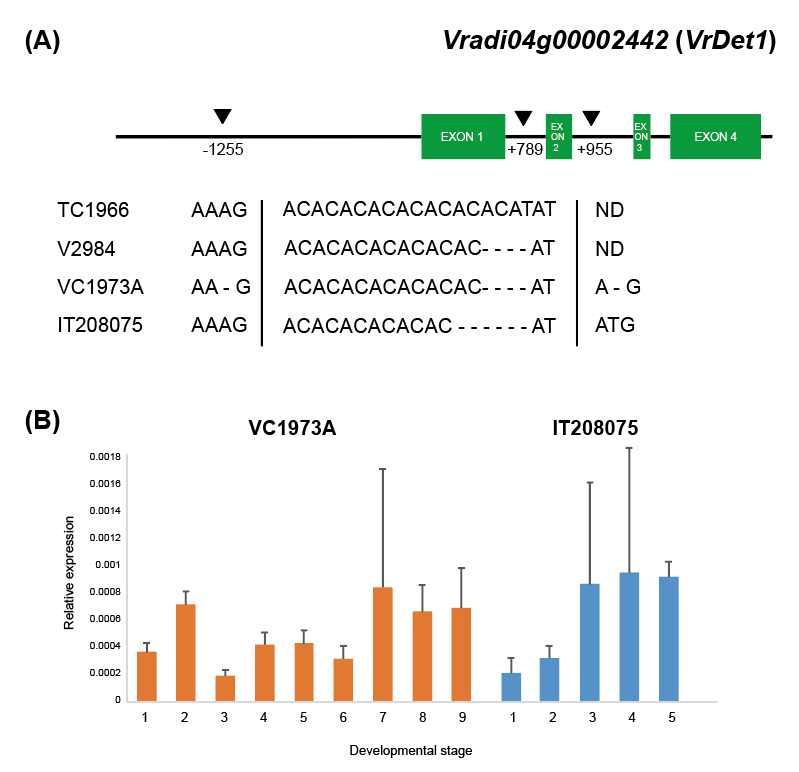


**Supplementary Figure 2.** Gene structure and expression level of *VrDet1*. **(A)** Indel variants found in the mungbean lines. Two insertion-deletion mutations between mapping parents was located in the upstream and second intron regions but the mutations were not detected in TC1966 and V2984 owing to unmapped information. Variation of simple repeat sequences was located in the first intron region between mungbean lines. All variation sites of the exon start site were indicated by black triangles. **(B)** Expression levels of *VrDet1* in shoot apical tissue from lines VC1973A and IT208075 at each developmental stage. ND: not detected owing to unmapped information.
